# Frequent Hybridisation Between Parapatric Lekking Bird‐of‐Paradise Species

**DOI:** 10.1111/mec.17780

**Published:** 2025-04-29

**Authors:** Filip Thörn, Ingo A. Müller, André E. R. Soares, Elizah Nagombi, Knud A. Jønsson, Mozes P. K. Blom, Martin Irestedt

**Affiliations:** ^1^ Department of Bioinformatics and Genetics Swedish Museum of Natural History Stockholm Sweden; ^2^ Department of Zoology Stockholm University Stockholm Sweden; ^3^ Leibniz Institut für Evolutions‐ und Biodiversitätsforschung, Museum für Naturkunde Berlin Germany; ^4^ National Bioinformatics Infrastructure Sweden, Science for Life Laboratory, Department of Medical Biochemistry and Microbiology Uppsala University Uppsala Sweden; ^5^ New Guinea Binatang Research Centre Madang Papua New Guinea; ^6^ Natural History Museum of Denmark Copenhagen Denmark

**Keywords:** birds‐of‐paradise, hybrid zone, hybridisation, introgression, lekking, museomics

## Abstract

Hybridisation is known to occur between a wide range of taxa, including species for which strong sexual selection has led to markedly different sexual phenotypes and lek‐mating behaviours. To what extent occasional hybridisation can overcome the reproductive barriers in such systems and, for example, lead to the establishment of hybrid zones is poorly known. In this study, we address this question by focusing on one of the most well‐known avian radiations in which sexual selection has resulted in an extraordinary assemblage of phenotypic diversity and lek‐mating behaviours: the birds‐of‐paradise (*Paradisaeidae*). We quantify the genome‐wide distribution of introgression and find multiple signals of recent and historical gene flow between and within two genera of birds‐of‐paradise, *Astrapia* and *Paradigalla*. In addition, we present the first empirical genomic indication of a putative hybrid zone between two lekking bird‐of‐paradise species that differ substantially in their sexually selected traits and behaviours. Our findings are consistent with the idea that behavioural and phenotypic traits may constitute weaker pre‐ and post‐zygotic barriers to gene flow than generally thought in lek‐mating species.

## 1 Introduction

The evolutionary consequences of hybridisation are a thriving research theme in evolutionary biology, which has commanded renewed attention with advances in whole genome sequencing (Twyford and Ennos 2012). While hybrids have sometimes been viewed as evolutionary dead ends, due to their purported reduced fitness, interspecific hybridisation is now frequently viewed as an evolutionary conduit in which adaptive or neutral variants can be exchanged and the reassembly of existing genetic variation may even facilitate adaptive radiation (Brumfield et al. 2001; Marques et al. 2019). However, as divergence between species increases, the evolution of pre‐ and post‐zygotic isolating mechanisms eventually often leads to decreased gene flow (Orr 2005). Pre‐zygotic barriers act before mating, that is, through behavioural (e.g., vocalisation, displays, or plumage traits) or mechanical (e.g., incompatible physiological structures) barriers (Henrich and Kalbe 2016; Jiggins et al. 2001), whereas post‐zygotic barriers prevent the formation of viable offspring after mating (i.e., genetic incompatibilities) (Dobzhansky 1936; Haldane 1922).

Hybrid zones serve as ideal study systems to investigate the contribution of pre‐ and post‐zygotic isolating mechanisms and how these can contribute to the cessation of gene flow between species. Hybrid zones are geographically defined regions where individuals from genetically distinct populations or species come into contact and hybridise (Barton and Hewitt 1985). The genomic characterisation of hybrid zone dynamics can be used to identify genomic regions that experience introgression or promote reproductive isolation (Peñalba et al. 2024). However, well‐separated species that come into secondary contact (i.e., sympatry) may collapse if hybrid fitness is high (Irwin and Schluter 2022) or if selection against hybrids is weak (Kearns et al. 2018), even when levels of hybridisation are low. Alternatively, a stable hybrid zone may be established and persist between distinct taxa for long periods of time (Poelstra et al. 2014). Such stable hybrid zones either persist through a balance between dispersal into the zone and selection against hybrids, that is, ‘tension zones’ (Barton and Hewitt 1985), or when hybrids have higher fitness than the parental forms in the environment of the hybrid zone, that is, ‘hybrid‐superiority hypothesis’ (Moore 1977). Hybrid zones involving species with strong sexual selection are of prime interest since they can elucidate the interplay between sexual preference, introgression and the emergence of reproductive isolation (Bennett et al. 2021). However, to date, few studies have characterised the genomic consequences of hybridisation and the possible existence of hybrid zones between lekking species (e.g., Brumfield et al. 2001), that constitute an extreme form of sexual selection. Here we address these questions by focusing on one of the most well‐known avian radiations where lekking has resulted in an extraordinary assemblage of phenotypic diversity, the birds‐of‐paradise (Passeriformes: Paradisaeidae). The birds‐of‐paradise include 45 species (Gill et al. 2024), of which the vast majority are endemic to New Guinea, with a few species found in Australia and on Indonesian islands.

**FIGURE 1:**
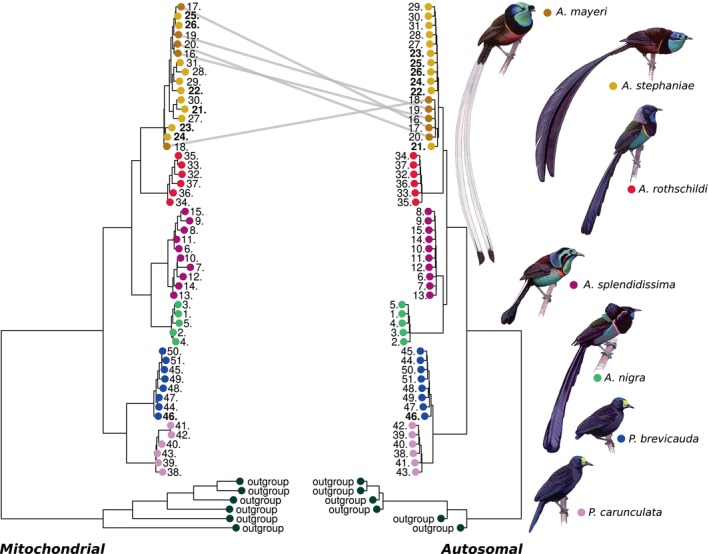
Comparison between mitochondrial tree (left) and autosomal summary coalescent tree for 5 kbp windows (right). Individuals (individuals 21, 22, 23, 24, 25 and 26) of *A. mayeri* and *A. stephaniae* show signs of recent hybridisation and are indicated in bold. Both phylogenetic trees are rooted with the clade *Lycocorax*, *Manucodia* and *Phonygammus* (black dots). Clades with full bootstrap support marked with 1 in mitochondrial phylogenetic tree. Clades marked with bootstrap/gene concordance factor/site concordance factor in the autosomal phylogenetic tree. Illustrations by Szabolcs Kókay.

Most species in the family of birds‐of‐paradise, Paradisaeidae, have developed elaborate plumage ornaments and complex mating behaviours through strong sexual selection (Ligon et al. 2018). Despite these apparent pre‐zygotic barriers, hybrid combinations between many sympatrically occurring bird‐of‐paradise species have been identified (Frith and Beehler 1998; Blom et al. 2024; Thörn et al. 2024). A recent study identified widespread ancient hybridisation across large parts of the birds‐of‐paradise tree (Blom et al. 2024) and a follow‐up study presented the first empirical evidence of backcrossing between deeply divergent contemporary bird‐of‐paradise species (Thörn et al. 2024), suggesting that pre‐ and post‐zygotic barriers may be more permeable than originally thought. To further examine how frequently introgression occurs in birds‐of‐paradise and whether cryptic contemporary hybrids exist, we apply museomics to study hybridisation in the reciprocally monophyletic sister‐genera *Astrapia* and *Paradigalla*. Members of these two genera have morphological similarities and have in the past been considered as members of a single genus (Dickson and Christidis 2014). Yet, the two genera differ significantly in both plumage, gross morphology and mating behaviours—members of *Astrapia* are more colourful, have considerably longer tails (Figure 1) and have leks in contrast to the two *Paradigalla* species. In spite of these differences, morphological hybrids between the two genera are known from the wild (Frith and Beehler 1998; Blom et al. 2024) and it has recently been shown that intergeneric hybrids between the two genera are able to backcross (Thörn et al. 2024). While the two genera have largely overlapping distributions, species belonging to the same genus generally have allopatric distributions preventing contemporary hybridisation. However, two species, *Astrapia mayeri* and *Astrapia stephaniae* , have partially overlapping distributions which coincide with one of only two geographic regions that may harbour a hybrid zone within the birds‐of‐paradise (Frith and Beehler 1998).

In the present study, we sequenced multiple individuals per species from the genera *Astrapia* and *Paradigalla* to investigate the frequency of hybridisation within and between the two genera. We hypothesise that: (1) large‐scale hybridisation patterns are related to biogeographical distributions, as it is well established that the orogeny of the New Guinean mountain ranges has affected speciation events in many of the island's present‐day bird fauna (Jønsson, Reeve, et al. 2019; Kennedy et al. 2022; Pujolar et al. 2022), and (2) that *Astrapia mayeri* and *Astrapia stephaniae* individuals in the regions where these two species co‐occur have distinctive genomic signatures of hybrid zones. We explore this unique system by quantifying the genome‐wide distribution of introgression and discuss these patterns in relation to the expected recombination rate and sexual selection.

## 2 Material and Methods

### 2.1 Species Information

**FIGURE 2:**
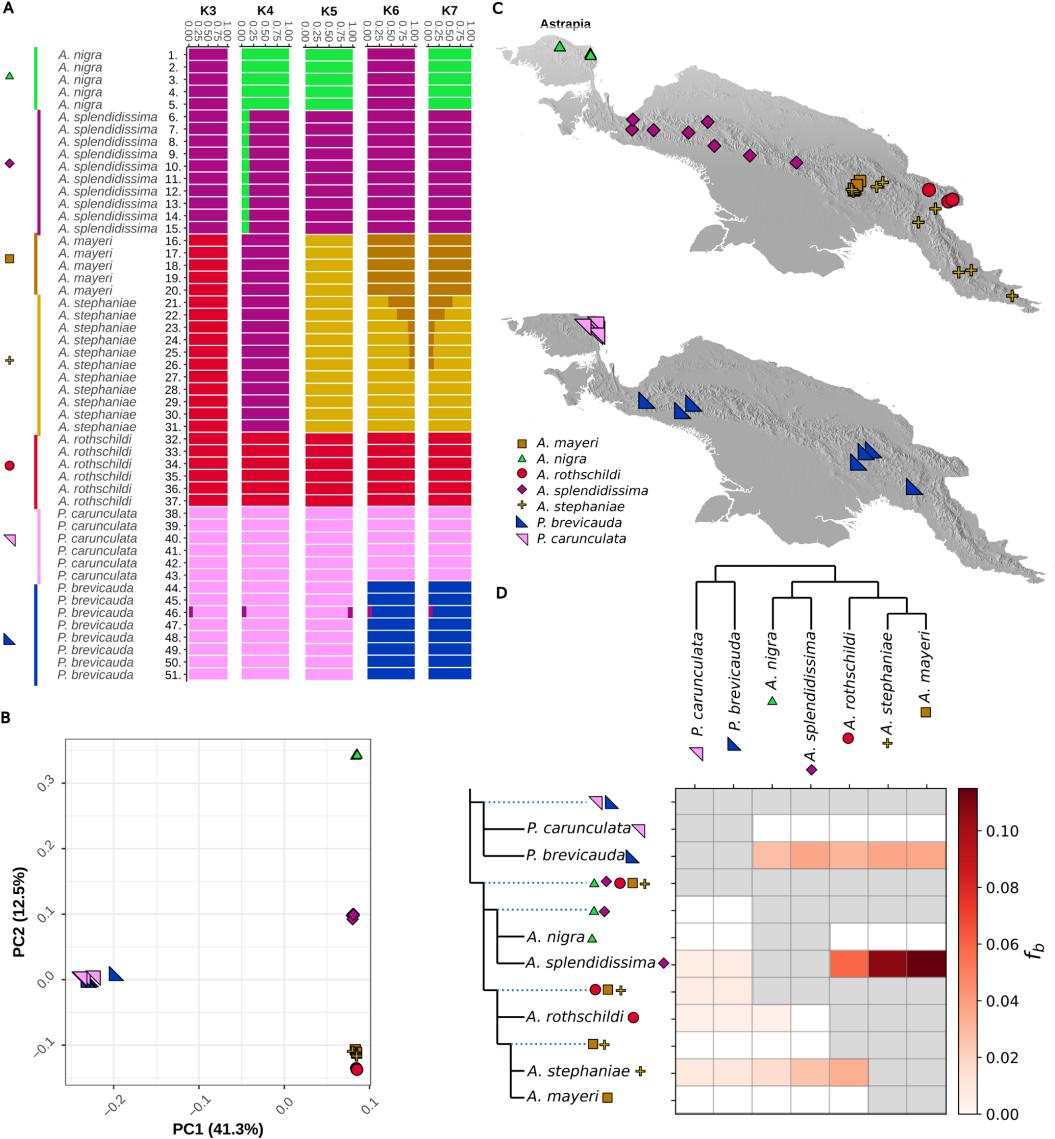
(A) Admixture for K3 to K7 using NGSadmix displaying signs of contemporary hybridisation. Sample identifiers are provided in Table S1. The samples of *A. mayeri* and *A. stephaniae* are ordered along a longitudinal gradient from west to east. (B) PCA of PC axis 1 and 2 calculated with PCAngsd displaying relationships between species. (C) Location of samples, higher resolution and individually marked in Figures S1 and S3. (D) Fbranch statistic is calculated using Dsuite indicating historical gene flow.

This study is based on re‐sequenced whole genome data from 51 samples of 7 ingroup taxa, of which 42 have been sequenced for this study, and six outgroup species that have been used to root the phylogenetic trees. For ingroup species, the sampling of individuals was selected to cover their geographical distribution to the extent possible, to account for putative population structure within species. The genus *Astrapia* includes five species: *Astrapia splendidissima* , *Astrapia mayeri* , *Astrapia stephaniae* , *Astrapia nigra* and *Astrapia rothschildi* . The first three species mate through classical lekking behaviour and display courtship behaviour in large groups. *A. nigra* and *A. rothschildi* , on the other hand, have exploded leks, where the courtship display is auditory rather than visual, so that males hear rather than see each other (Ligon et al. 2018). These two species are present on relatively isolated mountain ranges: *A. nigra* in the Arfak mountains in the northwest of New Guinea and *A. rothschildi* in the Huon mountains in the northeast of New Guinea. The remaining *Astrapia* species occupy different parts of the Central Mountain Range, which spans New Guinea from west to east. *A. splendidissima* occupies the western and central highlands, whereas *A. stephaniae* inhabits the eastern highlands. *A. mayeri* is a range‐restricted species present only in the eastern highlands, where it overlaps with *A. stephaniae* . The genus *Paradigalla*, which is the sister clade to *Astrapia*, includes two allopatrically distributed species: *Paradigalla brevicauda* (occupying the central mountain range of New Guinea) and *Paradigalla carunculata* (occupying the northwestern Bird's Head of New Guinea), both of which display solitarily (Ligon et al. 2018). Sample locations are plotted in Figure 2 and species distribution ranges in Figures S2 and S4. We sampled a minimum of five individuals per ingroup species and for species with wide distribution, up to 11 individuals have been included to cover their entire geographic distribution. Moreover, we included additional samples from the regions where two species overlap, that is, *A. splendidissima* , *A. stephaniae* and *P. brevicauda* . Most genomic data (*n* = 42) were re‐sequenced footpad samples from natural history collections. Genomic data for two samples were obtained from fresh tissue samples. Genomic data from an additional seven individuals were sourced from Blom et al. (2024). For detailed information on specimens included in the study see Table S1.

### 2.2 DNA Extractions, Library Preparations and Post‐Sequencing Treatment

DNA extractions and library preparations were carried out at the historical DNA lab at the Swedish Museum of Natural History and following the procedures presented in Irestedt et al. (2022). In short, DNA was extracted using the DNeasy Blood & Tissue Kits (QIAGEN) using the Irestedt et al. (2022) modifications to the manufacturer's protocol. Following DNA extractions, libraries were prepared using the Meyer and Kircher (2010) protocol with the modifications described in Irestedt et al. (2022). USER‐enzyme treatment was employed to decrease the effect of post‐mortem DNA damage on downstream analyses (Briggs et al. 2007). Following PCR amplifications and post‐PCR magnetic bead cleaning, libraries were pooled at equimolar DNA concentrations for sequencing in batches of 24 individuals and sequenced on the Illumina Nova‐seq 6000 platform (S4 flow cell, 100 bp pair end reads) at NGI SciLifeLab, Solna, aiming at a sequence read depth of 10×. The two fresh tissue samples were extracted using a KingFisher extraction kit and prepared and sequenced on an Illumina Nova‐seq 6000 platform (S4 flow cell, 150 bp paired end reads) at NGI SciLifeLab, Solna.

Following sequencing, sequencing libraries were evaluated using FastQC (v.0.11.8; Andrews 2010), treated with PCR‐deduplication (v.1.3.3; HTStream/hts_SuperDeduper; https://s4hts.github.io/HTStream/), adapter content trimming (Trimmomatic v.0.39; Bolger et al. 2014), merging of overlapping forward and reverse reads (PEAR v.0.9.11; Zhang et al. 2014), quality trimming (Trimmomatic v.0.39; Bolger et al. 2014) and removal of low‐complexity reads. All post‐sequencing treatment steps mentioned above were carried out using the nf‐polish pipeline with the default settings (https://github.com/MozesBlom/nf‐polish).

### 2.3 Mitochondrial Genome

Mitochondrial scaffolds were assembled with MitoBIM based on a random subset of 5 million polished read pairs using the *Lycocorax obiensis* mitochondrion scaffold (Peona et al. 2021) as a starting seed (v.1.9.1; Hahn et al. 2013). Using the MitoBIM assembled scaffold as a backbone, the entire read pool of polished DNA fragments was mapped against the reference to obtain summary statistics and detect signs of contamination through heterozygous positions. The process of reconstructing mitochondrial genomes was implemented in parallel for all individuals using nf_mito‐mania (https://github.com/FilipThorn/nf_mito‐mania).

An alignment of the assembled mitochondria was constructed with MAFFT using the Needleman‐Wunsch algorithm to align pairs (–globalpair) within 1000 iterations (v.7.407; Katoh et al. 2002). Species for the genera, *Lycocorax*, *Manucodia* and *Phonygammus*, were included in the mitochondria alignment to root the downstream phylogenetic tree, as these species belong to the monomorphic clade of birds‐of‐paradise that are sister to core birds‐of‐paradise to which the focal species belongs (Irestedt et al. 2009; Blom et al. 2024). The mitochondrial sequences from these species were obtained from Blom et al. (2024). A maximum likelihood phylogenetic tree was constructed from the alignment using RAxML‐NG with the GTR‐G substitution model and 100 bootstrap replicates (v.0.1.1; Kozlov et al. 2019). The mitochondrial phylogenetic tree was plotted with R (4.3.0) using the packages ggplot2 (v.3.4.2) and ggtree (v3.7.2; Yu et al. 2017).

### 2.4 Nuclear Mapping

The treated DNA fragments were mapped to chromosome‐spanning scaffolds using the *Lycocorax obiensis*, Paradisaeidae, (Peona et al. 2021) as the reference genome, which is a phylogenetic outgroup for all focal taxa in this study (Blom et al. 2024). Nuclear genomes were reconstructed by mapping the polished reads to the *L. obiensis* reference genome using bwa‐mem2 (v.2.2.1, Vasimuddin et al. 2019) and were implemented with nf‐umap (https://github.com/IngoMue/nf‐umap). Visual assessment of post‐mortem DNA damage was carried out with mapDamage2 (Jónsson et al. 2013).

### 2.5 Nuclear Phylogenetic Reconstruction

The autosomal phylogenetic tree was constructed using a whole genome phylogenetic inference pipeline; nf‐phylo (https://github.com/MozesBlom/nf‐phylo). Consensus sequences were constructed for each individual, based on hard called variants per individual using freebayes (v.1.3.1; Garrison and Marth 2012); heterozygous sites, low depth sites (3×), extreme high depth sites (100×) were masked, Multiple Nucleotide Polymorphisms (MNPs) were decomposed and indels were removed. Four different window sizes (1, 5, 10 and 20 kb) were sampled every 100 kb, a phylogeny was inferred for each window tree using IQtree2 (v.2.0.3; Minh, Schmidt, et al. 2020) and a summary‐coalescent species tree was inferred using ASTRAL3 (v5.7.8; Zhang et al. 2018). Each window was filtered and assessed for a range of criteria. Alignment columns were removed if more than 40% of individuals were missing for that position, individuals were removed with more than 50% missing data and windows were entirely filtered out if the length of the resulting window was below 50% of the original window length or if more than 20% of the individuals were missing. If windows were filtered out, the adjacent window was selected and evaluated for the same criteria. A gene tree was inferred for each filtered window using IQtree's and ModelFinderPlus to determine the best substitution model on a per‐window basis (Kalyaanamoorthy et al. 2017). We also used IQtree2 to reconstruct maximum likelihood phylogenetic trees by concatenating all autosomal windows for each different window size (1, 5, 10 and 20 kb), using the general time reversible model allowing for a proportion of invariable sites with a discrete gamma distribution with four categories (GTR + I + G). Finally, site and window concordance factors were calculated (Minh, Hahn, et al. 2020) and added for branch support in the resulting phylogenetic tree.

### 2.6 Species Delineation, Population Structure and Demographic History

The relationships between and within the different species were investigated with admixture and principal component analysis (PCA) based on genotype likelihoods. The genotype likelihoods were calculated with ANGSD using the settings: minMapQ = 20, minQ = 20, minMaf = 0.03, setMinDepthInd = 2, setMinDepth = 20 (v.0.933; Korneliussen et al. 2014). The genotype file was linkage pruned by keeping every 50th variant. Admixture proportions were estimated up to *k* = 7 with the tool NGSAdmix selecting the *K* value with the highest likelihood from 10 replications (v.0.933; Korneliussen et al. 2014) and PCA was calculated using PCAngsd (v.0.982; Meisner and Albrechtsen 2018). The workflow was implemented with nf‐GL_popstructure (https://github.com/FilipThorn/nf‐GL_popstructure). Plotting was done in R (v.4.3.0) using the packages ggplot2 (v.3.4.2).

Single nucleotide polymorphisms (SNP) were called in freebayes (v.1.3.1; Garrison and Marth 2012) using the joint call method, grouping individuals from the same species as a population prior. The *Lycocorax obiensis* genome was used as a reference and a minimum read quality of 20 was used. Vcftools (v.0.1.16; Danecek et al. 2021) was used to remove indels and filter out sites if they had a minor allele frequency below 0.03, a variant present in at least 90% of the population, individual sequencing depth below 2×, variant depth below 10× and above 75×. The resulting variant call file (VCF) was used to assess pairwise differentiation between all species with fixation indices (Fst) using the *Weir and Cockerham* method in vcftools for 50 K non‐overlapping windows. We excluded individuals that had indications of hybrid ancestry (based on admixture proportions for *k* = 7; Figure 2A) when assessing pairwise differentiation between the species. We refer to these individuals henceforth as recent hybrids.

We investigated gene flow between species using Dsuite's Dtrios and present the results using Fbranch (Malinsky et al. 2021). We excluded all recent hybrids and ran Dsuite with the freebayes produced VCF for all possible trio combinations using a species tree based on the phylogenetic reconstruction of the autosomes as a guide tree. *Lycocorax obiensis* and species from the genera *Manucodia* and *Phonygammus* were used as an outgroup in the P4 position to polarise ancestral and derived alleles.

We investigated the relationship between phylogenetic and geographic distance between all samples by correlating branch lengths and geographic distances. Pairwise distance in branch length between samples from the maximum likelihood concatenated autosomal phylogenetic tree for 5 kb window size (Figure S28) was plotted against euclidean geographic pairwise distances between samples. Pairwise distances were calculated in R with the ape package (v.5.7‐1; Paradis et al. 2004) for branch lengths and with the geodata package (v.0.5‐8; https://github.com/rspatial/geodata) for the geographic distances. Both measurements were standardised to one standard deviation and a mean of zero. Location data were taken from available meta‐data for each sample, most of which had been collected long before the invention of precise geolocation tools. As such, accuracy in precise location data cannot be guaranteed and species occupying very small areas were excluded (i.e., *A. nigra* , *A. rothschildi* and, *P. carunculata* ).

Using the Pairwise Sequentially Markovian Coalescent (PSMC), we obtained trends in population size for relative comparisons between species (Li and Durbin 2011). PSMC results are strongly influenced by the total coverage as well as the per‐site depth. When analysing low depth (< 8×) data, the amplitude of the PSMC curve decreases and leads to an underestimation of the effective population size (*N*_e_). The general shape of the PSMC curve also differs in low coverage samples depending on the per‐site depth threshold that is used (Nadachowska‐Brzyska et al. 2016). To reduce the risk of introduced variants, low depth and coverage affecting the interpretation of the PSMC analyses, we only selected one high coverage sample per species for these analyses (sequencing depth; 11×–23×), relative to all the samples included in the study. Further, we only selected individuals that had no indication of recent hybridisation in the admixture or PCA analyses. In addition to PSMC, we also calculated F_1_‐hybrid Pairwise Sequentially Markovian Coalescent (hPSMC; Cahill et al. 2016) to estimate the initial end of gene flow, that is, population divergence time, between all species combinations. The PSMC and hPSMC were run for 100 bootstraps and plotted in R (v.4.3.0) with the ggplot2 package (v.3.4.2). Samtools mpileup (v.1.2; Danecek et al. 2021) was used to generate the consensus scaffolds for all autosomes using the *Lycocorax obiensis* reference genome with a minimum depth of 8×, a maximum depth of 50×, and minimum quality score of 15. Parameters used for PSMC and hPSMC were ‐N25 ‐t15 ‐r6 ‐p “4 + 25*2 + 4 + 6” and a generation time of 8 years ( *Astrapia stephaniae* ; Bird et al. 2020) and mutation rate of 1.4e‐09 (Nadachowska‐Brzyska et al. 2016) were used when plotting.

### 2.7 Contemporary Hybrids

We performed additional analyses on the individuals identified as recent hybrids to assess how recently the hybridisation event occurred and to examine genome‐wide introgression patterns. The filtered joint called freebayes VCF was used to calculate ancestry informative markers (AIMs) for each of the recent hybrids identified from NGSadmix. The AIMs are identified based on Fst and have been used for confirmation of recent hybrids in past studies (Blom et al. 2024; Thörn et al. 2024). AIMs are obtained using vcftools weir‐*F*_ST_ between a hybrid that is to be investigated and two sets of parental populations used as a reference for the ancestral state. Sites that are fixed at different alleles between the two reference populations are then marked and genotyped in the hybrid individual. These sites are referred to as AIMs and plotted based on their parental identity in the hybrid. The relative ratio of heterozygous to homozygous AIMs will give an indication if the hybrid is a first‐generation hybrid, a later‐generation hybrid or backcross. We also calculated hybrid indices based on the ratio of parental alleles present in each hybrid (sum of counts of parent1 alleles from homozygote and heterozygous AIMs divided by the total number of alleles) and plotted it against interspecific heterozygosity (counts heterozygous AIMs divided by the total number of AIMs) (Valencia‐Montoya et al. 2020) to identify the direction of gene flow in backcrosses. We compiled the workflow of obtaining the AIMs and plotting them across the scaffolds and the hybrid indices calculations in the nextflow pipeline (https://github.com/FilipThorn/nf‐AIMs).

To investigate chromosomal regions that are more susceptible to gene flow, we looked at admixture patterns in hybrids across chromosomes by using a genotype likelihood‐based approach utilising NGSadmix (v.0.933; Korneliussen et al. 2014). In the AIMs analyses, we identified five hybrids that appeared to be backcrossing into *A. stephaniae* and were used to identify chromosomal regions experiencing higher introgression. We used five individuals of each species without hybrid content as a baseline and to control for ancestral polymorphisms through incomplete lineage sorting (ILS). Large chromosomes (> 40 Mbp) are divided into nine windows, intermediate chromosomes (> 20 and < 40 Mbp) are divided into seven windows, and micro chromosomes (< 20 Mbp) are divided into five windows, and NGSadmix is used to calculate admixture proportions for each window. NGSadmix is calculated in ten repetitions, and the repetition with the highest likelihood value is selected. If any individual in the reference panel exhibits admixture for the region, it is marked as containing ancestral polymorphisms, and incomplete lineage sorting (ILS) cannot be ruled out for that region. Since we are interested in regions being retained from the initial hybridisation event, we call admixture proportions so that 1 is 100% *A. mayeri* and 0 is 100% *A. stephaniae* . The median admixture proportion is calculated across all five hybrids in each window and plotted in ideograms with the regions painted with corresponding admixture proportion, where high proportions indicate high retained *A. mayeri* content (Method description; Figure S28). In addition, the admixture proportions for each window are plotted in boxplots for the different chromosome groups (micro, intermediate, and macro) to display introgression patterns relative to chromosomal window placements; windows marked for putative ILS were not included in these plots. The method was implemented in nextflow and is available at (https://github.com/FilipThorn/nf‐admixPainter). The analysis was also repeated for individual 46, using *Paradigalla* and *Astrapia* as the parental species.

## 3 Results

### 3.1 Sample Statistics

In total, 37 specimens from the genus *Astrapia* ( *A. nigra* : 5; *A. splendidissima* : 10; *A. rothschildi* : 6; *A. mayeri* : 5, and *A. stephaniae* : 11) and 14 specimens from the genus *Paradigalla* ( *P. carunculata* : 6, and *P. brevicauda* : 8) were sequenced. The average mapping rate of sequencing reads to the reference genome was 97.5%, and an average depth of coverage of 14.7× was obtained. We found no elevated excess of heterozygous positions in the mitochondria, indicating no cross‐contamination during library preparations. All raw reads generated for this study have been deposited at the European Nucleotide Archive (ENA), accession number PRJEB73831.

### 3.2 Species Delineation and Population Structure

Most of the seven recognised species segregated in the PCA plot (Figure 2B). PC axis 1 displays the difference between the genera *Astrapia* and *Paradigalla*, while PC axis 2 displays the difference within *Astrapia*. Of the *Astrapia* species, *A. nigra* , *A. splendidissima* and *A. rothschildi* form their own separate clusters along PC axis 2, while *A. mayeri* and *A. stephaniae* individuals cluster tightly together. The specimen (PbreX075320/ind 46) did not cluster with the other *P. brevicauda* individuals. This individual is a recent hybrid between the two genera *A. splendidissima* and *P. brevicauda* (described in detail in Thörn et al. 2024), which have backcrossed with *P. brevicauda* (Figures S7, S14, S16). Admixture barplots for *k* = 3 to *k* = 7, *K* = 7 had the highest support (Figure 2A), showing multiple individuals exhibiting hybrid content between *A. mayeri* and *A. stephaniae* (specimens 21, 22, 23, 24, 25, 26). We are using admixture proportion as a proxy for hybrid content, but as admixture proportions may be affected by continuous population divergence following patterns of isolation by distance, we confirmed the hybrid identity of these individuals using AIMs. Furthermore, from the pairwise species *F*_ST_ calculations (Figure S5), we detected the highest differences between the genera and the least differences between the species *A. mayeri* and *A. stephaniae* . Moreover, phylogenetic branch lengths generally increase within and between species as geographic distances increase (Figure S22), suggesting a geographical limitation to gene flow within and between species.

### 3.3 Phylogenetic Reconstructions

Excluding the recent hybrid (specimen 21), all species formed monophyletic clades across all different window sizes (1, 5, 10 and 20 kb) of the autosomal summary coalescent phylogenetic trees and in the mitochondrial phylogenetic tree, with the exception of *A. mayeri* and *A. stephaniae* that formed a mixed clade in the mitochondrial phylogenetic tree (Figure 1). All species clades had 100% bootstrap support with the exception of the *A. mayeri* and *A. stephaniae* clade in the mitochondrial phylogenetic tree. The site and window concordance factors differ a lot depending on the window size used for the summary coalescent phylogenetic trees; the largest window size (20 kb) received a relatively low level of discordance for species‐level bifurcations, while the smaller window sizes left these bifurcations with a high level of discordance. The bootstrap values, site and window concordance factors can be seen for all summary coalescent phylogenetic trees in Figures S23 and S26.

### 3.4 Demographic Histories

We used the hPSMC to estimate divergence times across the species tree. Based on these estimates, the two *Paradigalla* species diverged from a common ancestor around 2 million years ago. We estimate the divergence time between the two genera to be around 8 million years (dated species tree and hPSMC/PSMC; Figure S6). Furthermore, we found that species occurring on solitary mountain chains, such as the Huon in the east and Arfak in the west, have experienced substantial reductions in effective population size following the divergence (Figure S6).

Based on the Fbranch statistics, we identified three strong gene flow events between (1) *A. splendidissima* and *A. mayeri* , (2) *A. splendidissima* and *A. stephaniae* , and (3) *A. splendidissima* and *A. rothschildi* . We also find some weak gene flow events between *P. brevicauda* and all *Astrapia* species, and between *A. stephaniae* and *A. rothschildi* (Figure 2D). Fbranch statistics are a variation of a four‐taxon test and depend on the topology of the species tree to model historical gene flow. The model cannot model gene flow between sister‐taxa, which explains why some species combinations are not presented. It is important to note that all identified recent hybrids (specimens 21, 22, 23, 24, 25, 26 and 46) were excluded from the dataset prior to the analysis to avoid inflating the signal of introgression with recent hybrids.

### 3.5 Support for a Possible Hybrid Zone Between *A. mayeri* and *A. stephaniae*

**FIGURE 3:**
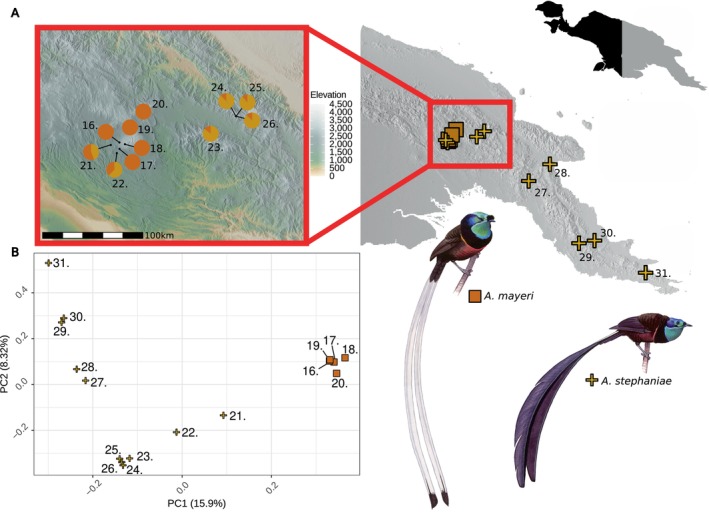
Locations and admixture proportions of *A. mayeri* and *A. stephaniae* samples. The samples are indexed after the order they appear in Figure 2. The sample locations of *A. mayeri* (square) and *A. stephaniae* (cross) with *A. stephaniae* samples indexed. (A) The putative hybrid zone is shown in the enlarged map and samples are plotted overlaying elevation at a resolution of 1 km^2^. The *A. stephaniae* samples excluded in the enlarged map (27, 28, 29, 30 and 31) did not show any indication of recent admixture. (B) Principal component analysis of *A. mayeri* (square) and *A. stephaniae* (cross). Illustrations of *A. stephaniae* and *A. mayeri* . painted by Szabolcs Kókay.

Evidence of ongoing hybridisation between *A. mayeri* and *A. stephaniae* was noticed in the admixture analyses (Figure 2A), mitochondrial phylogenetic tree (Figure 1) and pairwise *F*_ST_ patterns (Figure S5). To investigate this in more detail, we reduced the data set to repeat PCA and admixture analyses with only *A. mayeri* and *A. stephaniae* specimens (Figure 3). Among the six individuals with an admixed composition, the two *A. stephaniae* individuals (21, 22) most closely located to *A. mayeri* have the most admixed genomes. In the PCA, we found the recent hybrids situated between the parental species on PC1.

From the AIMs analyses (Figures S8 and S13), we uncovered that most of the recent hybrids (specimens 22, 23, 24, 25 and 26) had a higher proportion of homozygous *A. stephaniae* AIMs. Among these six admixed individuals, the four males had an excess of *A. stephaniae* homozygous genotype calls at the Z chromosome, and the two females both inherited an *A. stephaniae* Z chromosome. This finding suggests that they are the product of a hybridisation event between *A. mayeri* and *A. stephaniae* , followed by one or more backcrosses between the hybrid and *A. stephaniae* . For individual 21, we detected an equal proportion of AIMs that are homozygous for either of the parental species and a higher proportion of AIMs that are heterozygous. The proportion of homozygous AIMs was roughly 50% of the total number of AIMs, which suggests that this individual is an F2 individual, i.e., a result of a cross between two F1‐hybrids. This result was replicated in the triangle plot of the hybrid indices and interspecific heterozygosity (Figure S7).

**FIGURE 4:**
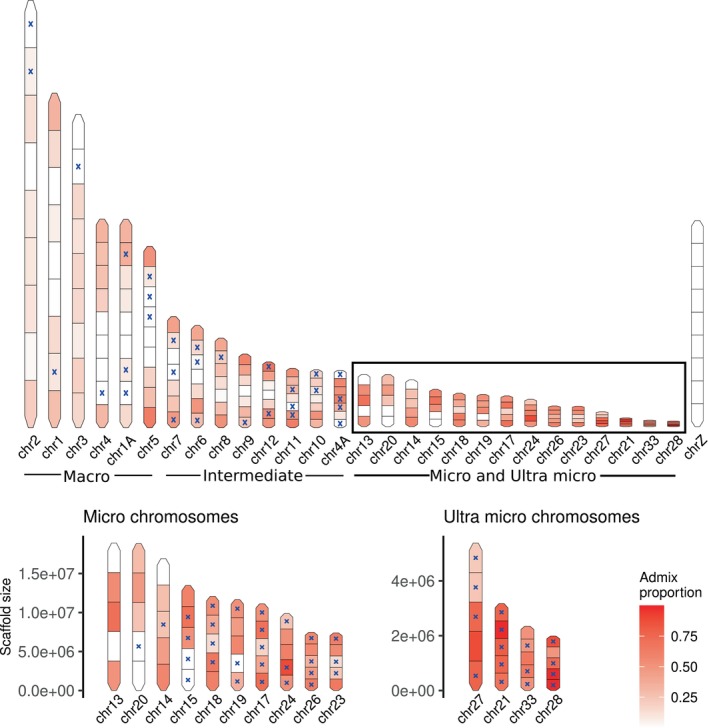
Admixture proportions across different regions of the chromosomes in hybrids between *A. mayeri* and *A. stephaniae* resulting from at least one backcross between a hybrid and *A. stephaniae* . High values indicate regions of high *A. mayeri* content, which has been preserved after backcrossing with *A. stephaniae* . A blue “x” indicates a specific region of the chromosomes where the parental species' reference panel display admixture between each other. These regions can therefore not be separated from regions of high likelihood of introgression and incomplete lineage sorting in the hybrids.

**FIGURE 5:**
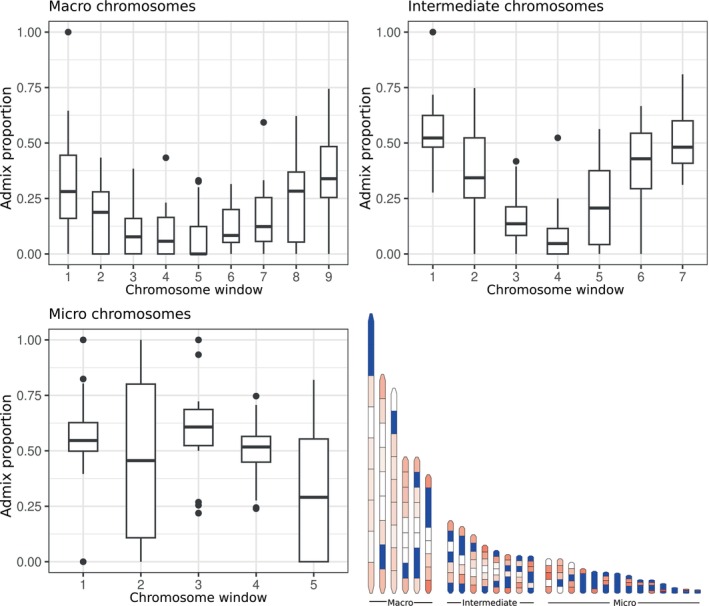
Introgression trends in windows distributed across chromosomes in backcrossing hybrids between *A. mayeri* and *A. stephaniae* . (A) Median admixture proportion across the five backcrossing hybrids in 9 windows distributed across large chromosomes (> 40Mbp), (B) Median admixture proportion across the five backcrossing hybrids in 7 windows distributed across intermediate chromosomes (< 40 Mbp and > 20Mbp), (C) Median admixture proportion across the five backcrossing hybrids in 5 windows distributed across micro chromosomes (< 20Mbp). (D) Chromosome cartoon displaying relative size of chromosomes. Admixture proportion in windows, white low and red high. Windows not separable form incomplete lineage sorting filled with blue and are excluded from the box plots.

We used the five recent hybrids (specimens 22, 23, 24, 25 and 26) that were identified as descendants from hybrids backcrossed into *A. stephaniae* , to detect regions more prone to introgression from *A. mayeri* across the chromosomes (Figure 4). We found no *A. mayeri* content in any block of the Z chromosome in contrast to a pattern of *A. mayeri* content in terminal blocks of the macro and most chromosomes of intermediate size (Figure 5). In the micro chromosomes we saw high *A. mayeri* content in most blocks. However, most of the blocks of the micro chromosomes do not segregate between the ancestral panel (marked with a blue x in Figure 4) and thus we cannot dismiss incomplete lineage sorting.

## 4 Discussion

In this study, we unravel complex patterns of contemporary and historical hybridisation within and between two genera of birds‐of‐paradise. We resolve phylogenetic relationships and identify gene flow between focal species. The clade comprising *Astrapia* and *Paradigalla* includes seven species confined to montane regions across the island of New Guinea. These species have markedly different plumages and exhibit significant variation in lekking behaviour. The signatures of hybridisation revealed herein, both within and between genera, are surprising as prominent phenotypical and behavioural differences are expected to act as strong pre‐zygotic barriers to gene flow. Focusing further on the putative hybrid zone between *A. mayeri* and *A. stephaniae* , we identify chromosomal hotspots that are more susceptible to introgression in the genomic landscape of the hybrids. Below we discuss the evolutionary significance of our results, which reveal how complex interactions between populations have shaped the genomic composition of contemporary populations and species.

### 4.1 Divergence and Biogeographic History

New Guinea has experienced major mountain uplifts in the last 10 million years (10 Mya). We find that the temporal estimates for the orogenies that led to the Central Mountain Range (10 Mya; Van Ufford and Cloos 2005) and the Huon mountains (4 Mya; Abbott et al. 1994; Van Ufford and Cloos 2005) coincide with a significant reduction in gene flow between the two genera, *Paradigalla* and *Astrapia* (7–9 Mya; hPSMC, Figure S6) and the divergence between *A. rothschildi* and its most recent common ancestor in the Central Mountain Range (3 Mya; hPSMC; Figure S6), respectively. Overall, our divergence time estimates suggest relatively recent splits around 2–3 Mya between species occurring in the outlying ranges and their sister‐taxa in the Central Range (hPSMC; Figure S6), which coincide with rapid and extensive mountain formation in New Guinea.

On New Guinea, the most common process of avian montane species formation is through recruitment from lowland species, which have been separated through vicariance events from mountain uplift (e.g., Pujolar et al. 2022; Kennedy et al. 2022; Reeve et al. 2023). Species occurring in isolated mountain ranges in this study ( *A. rothschildi* on Huon mountains and *A. nigra* and *P. carunculata* on the Arfak and Tamrau mountains) have all experienced large reductions in effective population sizes following their speciation events (PSMC; Figure S6). Thus, our results are in line with a scenario of gradual isolation and corresponding reduction of gene flow rather than dispersal between mountaintops that has been shown to be possible for certain New Guinean bird populations (Pujolar et al. 2022). Multiple other New Guinean bird species have *a* similar biogeographical pattern between sister‐taxa found in the Bird's Head Peninsula and in the Central Range (Irestedt et al. 2016, 2017; Jønsson, Blom, et al. 2019; Jønsson, Reeve, et al. 2019).

### 4.2 Historical and Contemporary Gene Flow

Our analyses reveal considerable historical gene flow between some of the species within *Astrapia* and between several *Astrapia* species and *P. brevicauda* (Figure 2D). Most of these gene flow combinations are geographically meaningful (i.e., gene flow is negatively correlated with geographical distance). However, some elevated levels of gene flow are unexpected (e.g., between *A. rothschildi* and *A. splendidissima* ). Yet, the Fbranch method used here is a variation of the four‐taxon test. Therefore, gene flow between sister taxa is not possible to detect, i.e., we cannot distinguish direct gene flow between geographically distant *A. rothschildi* and *A. splendidissima* from indirect gene flow through *A. mayeri* */stephaniae/rothschildi* that occupy areas in between *A. rothschildi* and *A. splendidissima* . Moreover, we also detect substantial gene flow between *P. brevicauda* and all *Astrapia* species in recent times. This finding is supported by the identified intergeneric backcross between *P. brevicauda* and *A. splendidissima* (Figures S7, S14, S15; see also Thörn et al. 2024). *P. brevicauda* and *A. splendidissima* co‐occur geographically in the Central Range, but replace each other at different elevations (Beehler and Pratt 2016).

### 4.3 Genomic Indication for a Possible Hybrid Zone Between Lekking Species

We identified six contemporary hybrids between *A. mayeri* and *A. stephaniae* based on the mitochondrial phylogenetic tree (Figure 1) and NGSadmix (nuclear genomic data; Figure 2A). All six hybrids were morphologically identified as *A. stephaniae* (four males and two females). Using AIMs and hybrid indices we show that all but one of these hybrids are recent backcrosses, as they share a greater proportion of AIMs with *A. stephaniae* (Figures S7 and S13). The exception to this is individual 21, which appears to be an F_2_‐hybrid (i.e., a cross between two F_1_ hybrids, Figure S7). The species *A. mayeri* and *A. stephaniae* occur in parapatry at the edge of either species' range (Figure 3). Hybrid individuals collected from this parapatric area (individuals 21 and 22) show considerably higher levels of admixture than hybrid individuals sampled outside the known distribution of *A. mayeri* (individuals 23, 24, 25 and 26), which suggests that there is gene flow from the zone of parapatry into that of allopatric *A. stephaniae* populations. At places where genetically distinct populations meet, a geographical hybrid zone may form in which frequent admixture occurs. Based on phenotypic data, previous studies (Frith and Beehler 1998) have hypothesized that a hybrid zone may be located in this region and here we provide the first genomic data that is consistent with this notion. The presence of both male and female backcrosses as well as an F_2_‐hybrid suggests that the accumulated differences between the species have not been sufficient to generate an asymmetric fitness reduction between the sexes as predicted by Haldane's rule (Haldane 1922). Additionally, all individuals sampled in the sympatric zone have some genomic signatures of recent hybridisation. This is an indication of frequent gene flow and likely weak pre‐zygotic barriers between the species, despite major differences in morphology and lek‐mating strategies. However, the identity of the Z chromosome of all hybrids is of *A. stephaniae* , which points to some restraints to gene flow. All analysed hybrids, except individual 21, experience asymmetrical gene flow from *A. mayeri* to *A. stephaniae* (Figures 3 and 4). Asymmetric gene flow is expected to move in the direction of the less‐fit population (Barton and Hewitt 1985), which may indicate a fitness disadvantage for hybrids at higher elevations where *A. mayeri* occurs (Beehler and Pratt 2016). Individual 21 is a hybrid between two F_1_‐hybrids (Figure S6), and therefore direct evidence that a female hybrid has been able to reproduce. Our analyses estimated an initial end of gene flow between *A. mayeri* and *A. stephaniae* to have taken place some 2 Mya (Figure S6), which strongly suggests that the putative hybrid zone is the result of secondary contact between the two species. However, the PSMC curves of the two species overlap up until 1Mya (Figure S6), which implies an extensive shared demographic history. Sampling of more individuals, particularly further east, is needed to further validate the presence of a hybrid zone and to characterise the width.

### 4.4 Chromosomal Introgression Patterns

Recombination rate and introgression are positively correlated with each other and vary across chromosomes (Martin et al. 2019). We have previously investigated chromosomal introgression patterns in hybrids using AIMs (Thörn et al. 2024). This approach identifies sites that are divergent between two species while fixed within each of the species. The parental assignments of these sites are then plotted across the chromosomes of a hybrid between the two populations. This method performed well for the intergeneric hybrid between *Paradigalla* and *Astrapia*, as these two genera have many fixed sites at different alleles (AIMs), which made it possible to identify introgressed tracts from *A. splendidissima* that have been retained along specific chromosomes (Figure S14; see also Thörn et al. 2024). However, when applying the AIMs method to the recent hybrids between *A. mayeri* and *A. stephaniae* (approx. 2 Mya hPSMC; Figure S6), very few sites were identified as AIMs (Figures S8–S13). Therefore, we developed a novel method for displaying introgression patterns across chromosomes in recent hybrids while controlling for incomplete lineage sorting (Figure 4). The method divides each chromosome into equal size blocks and estimates admixture proportions with NGSadmix (Korneliussen et al. 2014) for two reference populations and a single or multiple hybrids for two ancestral populations (*K* = 2). We expect the individuals in the two reference populations to have an admixture proportion of either extreme values (0 or 1). The admixture proportion of the hybrids will vary between the extremes, and the higher the value, the more alleles have been retained across a window through repeated backcrossing. Thus, the method can be used for hybrids between sister‐taxa. Genomic tracts in which incomplete lineage sorting (ILS) cannot be excluded are identified by having admixed signatures between the reference populations, indicating deep population structure. With this method, we identified terminal chromosomal windows to be more susceptible to introgression on large chromosomes (macro and intermediate). While all windows, regardless of chromosomal location, were susceptible to introgression on micro chromosomes, these windows were often not fixed between the parental species and, as such, not distinguishable from incomplete lineage sorting (ILS). Based on the recombination landscape of the zebra finch, it is expected that the ends of larger chromosomes are heavily biased towards more frequent recombination events and that the recombination rate is uniformly high across micro chromosomes (Backström et al. 2010), which is congruent with our results (Figures 4 and 5). Furthermore, all hybrids investigated with this method have a Z chromosome identity of *A. stephaniae* (with no traces of *A. mayeri* ). This is expected due to the low recombination rate of the Z chromosome (Backström et al. 2010) and gene flow being favoured in the direction from *A. mayeri* into *A. stephaniae* in the putative hybrid zone. Similarly, a previous study on ancestral hybridisation in birds‐of‐paradise found that the extent of introgression was also positively correlated with recombination rate, with more introgression towards the telomeres and limited introgression on the sex chromosomes (Blom et al. 2024).

Comparing the chromosomal patterns of *A. mayeri* and *A. stephaniae* (Figure 5) with the chromosomal patterns of *A. splendidissima* and *P. brevicauda* (Figure S15) shows that ILS is more frequent between *A. mayeri* and *A. stephaniae* . This is expected as these species have diverged much later than *A. splendidissima* and *P. brevicauda* . The higher susceptibility to gene flow towards the ends of larger and intermediate chromosomes is very similar between individual hybrids of *A. mayeri* and *A. stephaniae* . However, there is not a single hybrid genotype present in a hybrid zone but rather many different recombinants, something already noted by Barton and Hewitt (1985). While we show similar chromosomal introgression hotspots (Figures S17–S21), most likely mirroring recombination patterns, we do not believe that all hybrids between *A. stephaniae* and *A. mayeri* have the same genotype. Our method is simply not intended to detect finer differences in allele frequencies.

## 5 Conclusions

In this study, we find multiple signals of recent and historical gene flow between and within two sister genera of birds‐of‐paradise, known for prominent feather ornamentations and elaborate courtship rituals. We also present the first genomic analyses of a putative hybrid zone between two lekking species of birds‐of‐paradise as suggested from phenotypic evidence. We identify six contemporary hybrids, of which five have genome‐wide signals of asymmetric gene flow indicating a bias in successful matings in one direction. Furthermore, we present a novel method for identifying chromosomal regions that are more susceptible to gene flow in recent hybrids between sister taxa. Our findings of recurrent historical and contemporary hybridisation are evidence of incomplete reproductive barriers that still allow for gene flow between lekking bird‐of‐paradise species. We speculate that post‐zygotic reproductive barriers in lek‐mating systems may be weaker than generally thought.

## Author Contributions

M.I., M.P.K.B., and F.T. conceived the study. K.A.J. and E.N. conducted the fieldwork. F.T. and M.I. performed all laboratory work. F.T. carried out all data processing, analyses, and plotting, with input from A.E.R.S., I.A.M., and M.P.K.B. The first draft of the manuscript were drafted by F.T., and all authors contributed with comments and changes for the final version.

## Ethics Statement

This research complies with all relevant ethical regulations in Papua New Guinea. The PNG National Museum and Art gallery and the Conservation and Environment Protection Authority (CEPA) of Papua New Guinea provided research permits (99902749307 to K.A.J.) and export permits (CITES 019067).

## Conflicts of Interest

The authors declare no conflicts of interest.

## Supporting information


Table S1

Data S1

## Data Availability

All raw reads generated for this study have been deposited at the European Nucleotide Archive (ENA), accession number PRJEB73831. The files for the autosomal summary‐coalescent phylogenetic trees, the autosomal concatenated phylogenetic trees, the whole mitochondrial genome alignment and the mitochondrial phylogenetic tree files are uploaded to Dryad (https://doi.org/10.5061/dryad.rn8pk0pnt).

## References

[mec17780-bib-0001] Abbott, L. D. , E. A. Silver , P. R. Thompson , M. V. Filewicz , C. Schneider , and R. Abdoerrias . 1994. “Stratigraphic Constraints on the Development and Timing of Arc‐Continent Collision in Northern Papua New Guinea.” Journal of Sedimentary Research 64, no. 2b: 169–183. 10.1306/D4267F82-2B26-11D7-8648000102C1865D.

[mec17780-bib-0002] Andrews, S. 2010. “FastQC: A Quality Control Tool for High Throughput Sequence Data.” [Online]. http://www.bioinformatics.babraham.ac.uk/projects/fastqc/.

[mec17780-bib-0003] Backström, N. , W. Forstmeier , H. Schielzeth , et al. 2010. “The Recombination Landscape of the Zebra Finch *Taeniopygia guttata* Genome.” Genome Research 20, no. 4: 485–495. 10.1101/gr.101410.109. 20357052 PMC2847751

[mec17780-bib-0004] Barton, N. H. , and G. M. Hewitt . 1985. “Analysis of Hybrid Zones.” Annual Review of Ecology and Systematics 16, no. 1: 113–148. 10.1146/annurev.es.16.110185.000553.

[mec17780-bib-0005] Beehler, B. M. , and T. K. Pratt . 2016. Birds of New Guinea: Distribution, Taxonomy, and Systematics (REV‐Revised). Princeton University Press. https://www.jstor.org/stable/j.ctt17xr52d.

[mec17780-bib-0006] Bennett, K. F. P. , H. C. Lim , and M. J. Braun . 2021. “Sexual Selection and Introgression in Avian Hybrid Zones: Spotlight on Manacus.” Integrative and Comparative Biology 61, no. 4: 1291–1309. 10.1093/icb/icab135. 34128981

[mec17780-bib-0007] Bird, J. P. , R. Martin , H. R. Akçakaya , et al. 2020. “Generation Lengths of the World's Birds and Their Implications for Extinction Risk.” Conservation Biology 34, no. 5: 1252–1261. 10.1111/cobi.13486. 32058610

[mec17780-bib-0008] Blom, M. P. K. , V. Peona , S. Prost , et al. 2024. “Hybridization in Birds‐of‐Paradise: Widespread Ancestral Gene Flow Despite Strong Sexual Selection in a Lek‐Mating System.” iScience 27, no. 7: 110300. 10.1016/j.isci.2024.110300. 39055907 PMC11269930

[mec17780-bib-0009] Bolger, A. M. , M. Lohse , and B. Usadel . 2014. “Trimmomatic: A Flexible Trimmer for Illumina Sequence Data.” Bioinformatics 30, no. 15: 2114–2120. 10.1093/bioinformatics/btu170. 24695404 PMC4103590

[mec17780-bib-0010] Briggs, A. W. , U. Stenzel , P. L. F. Johnson , et al. 2007. “Patterns of Damage in Genomic DNA Sequences From a Neandertal.” Proceedings of the National Academy of Sciences of the United States of America 104, no. 37: 14616–14621. 10.1073/pnas.0704665104. 17715061 PMC1976210

[mec17780-bib-0011] Brumfield, R. T. , R. W. Jernigan , D. B. McDonald , and M. J. Braun . 2001. “Evolutionary Implications of Divergent Clines in an Avian (*Manacus aves*) Hybrid Zone.” Evolution 55, no. 10: 2070–2087. 10.1554/0014-3820(2001)055[2070:EIODCI]2.0.CO;2. 11761066

[mec17780-bib-0012] Cahill, J. A. , A. E. R. Soares , R. E. Green , and B. Shapiro . 2016. “Inferring Species Divergence Times Using Pairwise Sequential Markovian Coalescent Modelling and Low‐Coverage Genomic Data.” Philosophical Transactions of the Royal Society, B: Biological Sciences 371, no. 1699: 20150138. 10.1098/rstb.2015.0138. PMC4920339 27325835

[mec17780-bib-0013] Danecek, P. , J. K. Bonfield , J. Liddle , et al. 2021. “Twelve Years of SAMtools and BCFtools.” GigaScience 10, no. 2: giab008. 10.1093/gigascience/giab008. 33590861 PMC7931819

[mec17780-bib-0062] Dickson, E. C. , and L. Christidis , eds. 2014. The Howard and Moore Complete Checklist of the Birds of the World. Vol. 2. 4th ed. Passerines. Aves Press.

[mec17780-bib-0014] Dobzhansky, T. 1936. “Studies on Hybrid Sterility. II. Localization of Sterility Factors in *Drosophila pseudoobscura* Hybrids.” Genetics 21, no. 2: 113–135. 17246786 10.1093/genetics/21.2.113 PMC1208664

[mec17780-bib-0015] Frith, C. B. , and B. M. Beehler . 1998. Birds of Paradise: Paradisaeidae. OUP Oxford.

[mec17780-bib-0016] Garrison, E. , and G. Marth . 2012. “Haplotype‐Based Variant Detection From Short‐Read Sequencing.” arXiv. 10.48550/arXiv.1207.3907.

[mec17780-bib-0017] Gill, F. , D. Donsker , and P. Rasmussen . 2024. “IOC World Bird List (v14.2).” 10.14344/IOC.ML.14.1.

[mec17780-bib-0018] Hahn, C. , L. Bachmann , and B. Chevreux . 2013. “Reconstructing Mitochondrial Genomes Directly From Genomic Next‐Generation Sequencing Reads—A Baiting and Iterative Mapping Approach.” Nucleic Acids Research 41, no. 13: e129. 10.1093/nar/gkt371. 23661685 PMC3711436

[mec17780-bib-0019] Haldane, J. B. S. 1922. “Sex Ratio and Unisexual Sterility in Hybrid Animals.” Journal of Genetics 12, no. 2: 101–109. 10.1007/BF02983075.

[mec17780-bib-0020] Henrich, T. , and M. Kalbe . 2016. “The Role of Prezygotic Isolation Mechanisms in the Divergence of Two Parasite Species.” BMC Evolutionary Biology 16, no. 1: 245. 10.1186/s12862-016-0799-5. 27829374 PMC5103353

[mec17780-bib-0021] Irestedt, M. , H. Batalha‐Filho , P. G. P. Ericson , L. Christidis , and R. Schodde . 2017. “Phylogeny, Biogeography and Taxonomic Consequences in a Bird‐Of‐Paradise Species Complex, Lophorina–Ptiloris (Aves: Paradisaeidae).” Zoological Journal of the Linnean Society 181, no. 2: 439–470. 10.1093/zoolinnean/zlx004.

[mec17780-bib-0022] Irestedt, M. , H. Batalha‐Filho , C. S. Roselaar , L. Christidis , and P. G. P. Ericson . 2016. “Contrasting Phylogeographic Signatures in Two Australo‐Papuan Bowerbird Species Complexes (Aves: Ailuroedus).” Zoologica Scripta 45, no. 4: 365–379. 10.1111/zsc.12163.

[mec17780-bib-0023] Irestedt, M. , K. A. Jønsson , J. Fjeldså , L. Christidis , and P. G. Ericson . 2009. “An Unexpectedly Long History of Sexual Selection in Birds‐of‐Paradise.” BMC Evolutionary Biology 9, no. 1: 235. 10.1186/1471-2148-9-235. 19758445 PMC2755009

[mec17780-bib-0024] Irestedt, M. , F. Thörn , I. A. Müller , K. A. Jønsson , P. G. P. Ericson , and M. P. K. Blom . 2022. “A Guide to Avian Museomics: Insights Gained From Resequencing Hundreds of Avian Study Skins.” Molecular Ecology Resources 22, no. 7: 2672–2684. 10.1111/1755-0998.13660. 35661418 PMC9542604

[mec17780-bib-0025] Irwin, D. , and D. Schluter . 2022. “Hybridization and the Coexistence of Species.” American Naturalist 200, no. 3: E93–E109. 10.1086/720365. 35977784

[mec17780-bib-0026] Jiggins, C. D. , R. E. Naisbit , R. L. Coe , and J. Mallet . 2001. “Reproductive Isolation Caused by Colour Pattern Mimicry.” Nature 411: 6835. 10.1038/35077075. 11357131

[mec17780-bib-0027] Jónsson, H. , A. Ginolhac , M. Schubert , P. L. F. Johnson , and L. Orlando . 2013. “mapDamage2.0: Fast Approximate Bayesian Estimates of Ancient DNA Damage Parameters.” Bioinformatics 29, no. 13: 1682–1684. 10.1093/bioinformatics/btt193. 23613487 PMC3694634

[mec17780-bib-0028] Jønsson, K. A. , M. P. K. Blom , P. Z. Marki , et al. 2019. “Complete Subspecies‐Level Phylogeny of the Oriolidae (Aves: Passeriformes): Out of Australasia and Return.” Molecular Phylogenetics and Evolution 137: 200–209. 10.1016/j.ympev.2019.03.015. 30914395

[mec17780-bib-0029] Jønsson, K. A. , A. H. Reeve , M. P. K. Blom , M. Irestedt , and P. Z. Marki . 2019. “Unrecognised (Species) Diversity in New Guinean Passerine Birds.” Emu ‐ Austral Ornithology 119, no. 3: 233–241. 10.1080/01584197.2019.1581033.

[mec17780-bib-0030] Kalyaanamoorthy, S. , B. Q. Minh , T. K. Wong , A. von Haeseler , and L. S. Jermiin . 2017. “ModelFinder: Fast Model Selection for Accurate Phylogenetic Estimates.” Nature Methods 14, no. 6: 587–589. 10.1038/nmeth.4285. 28481363 PMC5453245

[mec17780-bib-0031] Katoh, K. , K. Misawa , K. Kuma , and T. Miyata . 2002. “MAFFT: A Novel Method for Rapid Multiple Sequence Alignment Based on Fast Fourier Transform.” Nucleic Acids Research 30, no. 14: 3059–3066. 10.1093/nar/gkf436. 12136088 PMC135756

[mec17780-bib-0032] Kearns, A. M. , M. Restani , I. Szabo , et al. 2018. “Genomic Evidence of Speciation Reversal in Ravens.” Nature Communications 9, no. 1: 906. 10.1038/s41467-018-03294-w. PMC5834606 29500409

[mec17780-bib-0033] Kennedy, J. D. , P. Z. Marki , A. H. Reeve , et al. 2022. “Diversification and Community Assembly of the World's Largest Tropical Island.” Global Ecology and Biogeography 31, no. 6: 1078–1089. 10.1111/geb.13484.

[mec17780-bib-0034] Korneliussen, T. S. , A. Albrechtsen , and R. Nielsen . 2014. “ANGSD: Analysis of Next Generation Sequencing Data.” BMC Bioinformatics 15, no. 1: 356. 10.1186/s12859-014-0356-4. 25420514 PMC4248462

[mec17780-bib-0035] Kozlov, A. M. , D. Darriba , T. Flouri , B. Morel , and A. Stamatakis . 2019. “RAxML‐NG: A Fast, Scalable and User‐Friendly Tool for Maximum Likelihood Phylogenetic Inference.” Bioinformatics 35, no. 21: 4453–4455. 10.1093/bioinformatics/btz305. 31070718 PMC6821337

[mec17780-bib-0036] Li, H. , and R. Durbin . 2011. “Inference of Human Population History From Individual Whole‐Genome Sequences.” Nature 475, no. 7357: 493–496. 10.1038/nature10231. 21753753 PMC3154645

[mec17780-bib-0037] Ligon, R. A. , C. D. Diaz , J. L. Morano , et al. 2018. “Evolution of Correlated Complexity in the Radically Different Courtship Signals of Birds‐of‐Paradise.” PLoS Biology 16, no. 11: e2006962. 10.1371/journal.pbio.2006962. 30457985 PMC6245505

[mec17780-bib-0038] Malinsky, M. , M. Matschiner , and H. Svardal . 2021. “Dsuite—Fast D‐Statistics and Related Admixture Evidence From VCF Files.” Molecular Ecology Resources 21, no. 2: 584–595. 10.1111/1755-0998.13265. 33012121 PMC7116594

[mec17780-bib-0039] Marques, D. A. , K. Lucek , V. C. Sousa , L. Excoffier , and O. Seehausen . 2019. “Admixture Between Old Lineages Facilitated Contemporary Ecological Speciation in Lake Constance Stickleback.” Nature Communications 10, no. 1: 4240. 10.1038/s41467-019-12182-w. PMC6751218 31534121

[mec17780-bib-0040] Martin, S. H. , J. W. Davey , C. Salazar , and C. D. Jiggins . 2019. “Recombination Rate Variation Shapes Barriers to Introgression Across Butterfly Genomes.” PLoS Biology 17, no. 2: e2006288. 10.1371/journal.pbio.2006288. 30730876 PMC6366726

[mec17780-bib-0041] Meisner, J. , and A. Albrechtsen . 2018. “Inferring Population Structure and Admixture Proportions in Low‐Depth NGS Data.” Genetics 210, no. 2: 719–731. 10.1534/genetics.118.301336. 30131346 PMC6216594

[mec17780-bib-0042] Meyer, M. , and M. Kircher . 2010. “Illumina Sequencing Library Preparation for Highly Multiplexed Target Capture and Sequencing.” Cold Spring Harbor Protocols 2010, no. 6: pdb.prot5448. 10.1101/pdb.prot5448. 20516186

[mec17780-bib-0043] Minh, B. Q. , M. W. Hahn , and R. Lanfear . 2020. “New Methods to Calculate Concordance Factors for Phylogenomic Datasets.” Molecular Biology and Evolution 37, no. 9: 2727–2733. 10.1093/molbev/msaa106. 32365179 PMC7475031

[mec17780-bib-0044] Minh, B. Q. , H. A. Schmidt , O. Chernomor , et al. 2020. “IQ‐TREE 2: New Models and Efficient Methods for Phylogenetic Inference in the Genomic Era.” Molecular Biology and Evolution 37, no. 5: 1530–1534. 10.1093/molbev/msaa015. 32011700 PMC7182206

[mec17780-bib-0045] Moore, W. S. 1977. “An Evaluation of Narrow Hybrid Zones in Vertebrates.” Quarterly Review of Biology 52, no. 3: 263–277. 10.1086/409995.

[mec17780-bib-0046] Nadachowska‐Brzyska, K. , R. Burri , L. Smeds , and H. Ellegren . 2016. “PSMC Analysis of Effective Population Sizes in Molecular Ecology and Its Application to Black‐And‐White Ficedula Flycatchers.” Molecular Ecology 25, no. 5: 1058–1072. 10.1111/mec.13540. 26797914 PMC4793928

[mec17780-bib-0047] Orr, H. A. 2005. “The Genetic Basis of Reproductive Isolation: Insights From Drosophila.” Proceedings of the National Academy of Sciences of the United States of America 102, no. Suppl_1: 6522–6526. 10.1073/pnas.0501893102. 15851676 PMC1131866

[mec17780-bib-0048] Paradis, E. , J. Claude , and K. Strimmer . 2004. “APE: Analyses of Phylogenetics and Evolution in R Language.” Bioinformatics 20, no. 2: 289–290. 10.1093/bioinformatics/btg412. 14734327

[mec17780-bib-0049] Peñalba, J. V. , A. Runemark , J. I. Meier , et al. 2024. “The Role of Hybridization in Species Formation and Persistence.” Cold Spring Harbor Perspectives in Biology 16, no. 12: a041445. 10.1101/cshperspect.a041445. 38438186 PMC11610762

[mec17780-bib-0050] Peona, V. , M. P. K. Blom , L. Xu , et al. 2021. “Identifying the Causes and Consequences of Assembly Gaps Using a Multiplatform Genome Assembly of a Bird‐Of‐Paradise.” Molecular Ecology Resources 21, no. 1: 263–286. 10.1111/1755-0998.13252. 32937018 PMC7757076

[mec17780-bib-0051] Poelstra, J. W. , N. Vijay , C. M. Bossu , et al. 2014. “The Genomic Landscape Underlying Phenotypic Integrity in the Face of Gene Flow in Crows.” Science 344, no. 6190: 1410–1414. 10.1126/science.1253226. 24948738

[mec17780-bib-0052] Pujolar, J. M. , M. P. K. Blom , A. H. Reeve , et al. 2022. “The Formation of Avian Montane Diversity Across Barriers and Along Elevational Gradients.” Nature Communications 13, no. 1: 268. 10.1038/s41467-021-27858-5. PMC8755808 35022441

[mec17780-bib-0053] Reeve, A. H. , J. D. Kennedy , J. M. Pujolar , et al. 2023. “The Formation of the Indo‐Pacific Montane Avifauna.” Nature Communications 14: 8215. 10.1038/s41467-023-43964-y PMC10713610 38081809

[mec17780-bib-0054] Thörn, F. , A. E. R. Soares , I. A. Müller , et al. 2024. “Contemporary Intergeneric Hybridization and Backcrossing Among Birds‐of‐Paradise.” Evolution Letters 8, no. 5: qrae023. 10.1093/evlett/qrae023. PMC11424083 39328285

[mec17780-bib-0055] Twyford, A. D. , and R. A. Ennos . 2012. “Next‐Generation Hybridization and Introgression.” Heredity 108, no. 3: 179–189. 10.1038/hdy.2011.68. 21897439 PMC3282392

[mec17780-bib-0056] Valencia‐Montoya, W. A. , S. Elfekih , H. L. North , et al. 2020. “Adaptive Introgression Across Semipermeable Species Boundaries Between Local *Helicoverpa zea* and Invasive *Helicoverpa armigera* Moths.” Molecular Biology and Evolution 37, no. 9: 2568–2583. 10.1093/molbev/msaa108. 32348505 PMC7475041

[mec17780-bib-0057] Van Ufford, A. Q. , and M. Cloos . 2005. “Cenozoic Tectonics of New Guinea.” AAPG Bulletin 89, no. 1: 119–140. 10.1306/08300403073.

[mec17780-bib-0058] Vasimuddin, M. , S. Misra , H. Li , and S. Aluru . 2019. Efficient Architecture‐Aware Acceleration of BWA‐MEM for Multicore Systems. 2019 IEEE International Parallel and Distributed Processing Symposium (IPDPS), 314–324. 10.1109/IPDPS.2019.00041.

[mec17780-bib-0059] Yu, G. , D. K. Smith , H. Zhu , Y. Guan , and T. T. Y. Lam . 2017. “ggtree: An R Package for Visualization and Annotation of Phylogenetic Trees With Their Covariates and Other Associated Data.” Methods in Ecology and Evolution 8, no. 1: 28–36. 10.1111/2041-210X.12628.

[mec17780-bib-0060] Zhang, C. , M. Rabiee , E. Sayyari , and S. Mirarab . 2018. “ASTRAL‐III: Polynomial Time Species Tree Reconstruction From Partially Resolved Gene Trees.” BMC Bioinformatics 19, no. 6: 153. 10.1186/s12859-018-2129-y. 29745866 PMC5998893

[mec17780-bib-0061] Zhang, J. , K. Kobert , T. Flouri , and A. Stamatakis . 2014. “PEAR: A Fast and Accurate Illumina Paired‐End reAd mergeR.” Bioinformatics 30, no. 5: 614–620. 10.1093/bioinformatics/btt593. 24142950 PMC3933873

