# Supplementary material for: Frequent Hybridisation Between Parapatric Lekking Bird‐of‐Paradise Species
Source: Mol Ecol. 2025 Apr 29;34(11):e17780. doi: 10.1111/mec.17780 (PMC12100584; doi:10.1111/mec.17780)
Supplement: Supplementary file 2 — Data S1 [file MEC-34-e17780-s002.pdf]

Supplement information for:

Frequent hybridisation between parapatric lekking bird-of-paradise species

Filip Thörn, Ingo A. Müller, André E. R. Soares, Elizah Nagombi, Knud A. Jønsson, Mozes P. K. Blom and Martin Irestedt

Table of contents	**Page**
Sample locations and species distributions	2-4
Pairwise Fst	5
Divergence time estimates	6
Triangle plot	7
Ancestry informative markers: *Astrapia mayeri* and *Astrapia stephaniae*	8-13
Ancestry informative markers: *Astrapia splendidissima* and *Paradigalla brevicauda*	14
AncestryPainter: *Astrapia splendidissima* and *Paradigalla brevicauda*	15-16
AncestryPainter: Individual chromosomal patterns for *Astrapia mayeri* and *Astrapia stephaniae*	17-21
Correlation between geographic distance and branch lengths	22
Autosomal phylogenies at different window sizes	23-27
AncestryPainter: step by step	28
Supplemental table text	29

Sample locations and species distributions

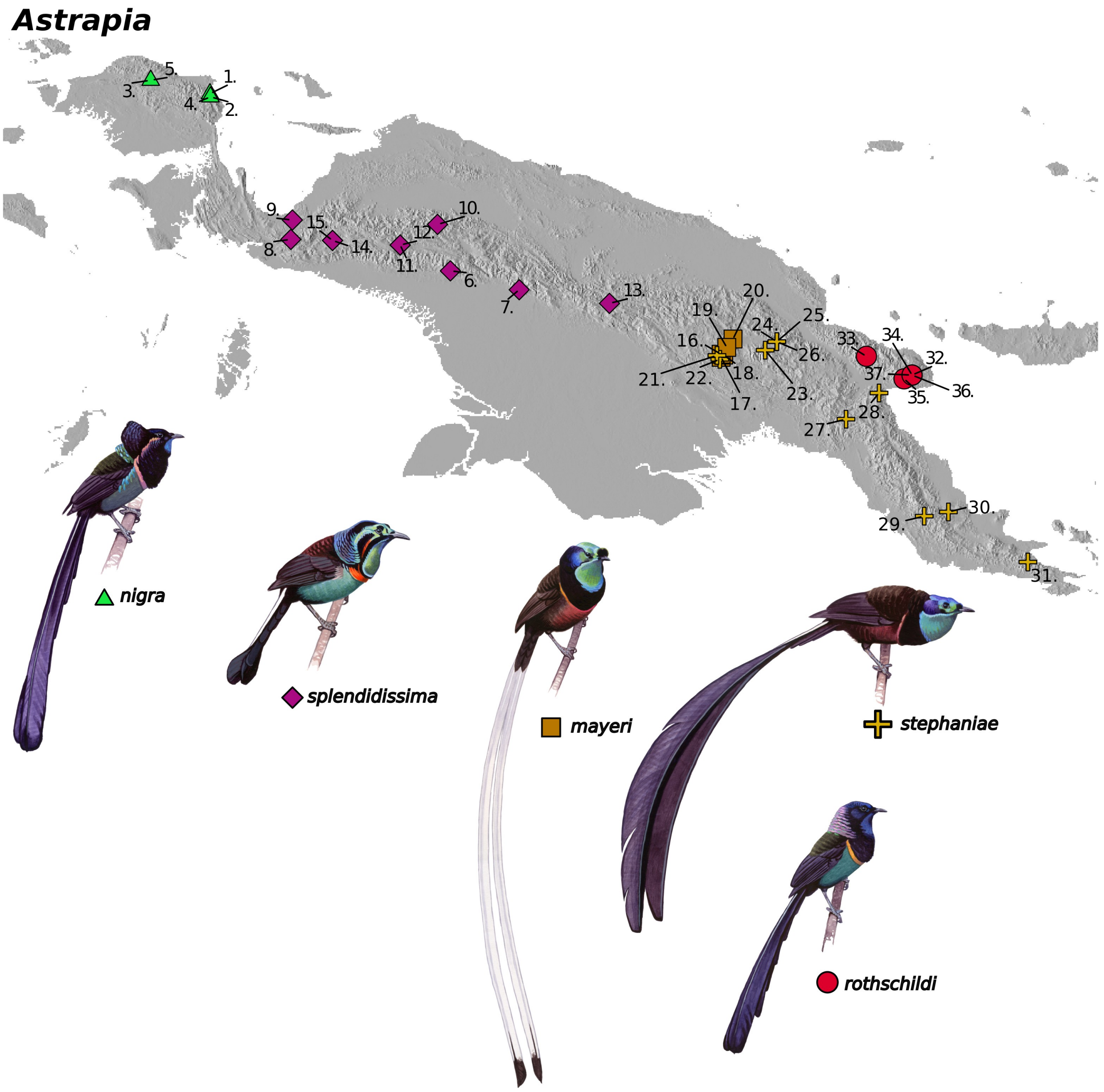

Figure S1. Sample locations of all *Astrapia* individuals. The numbering matches the order of the samples in figure 2.

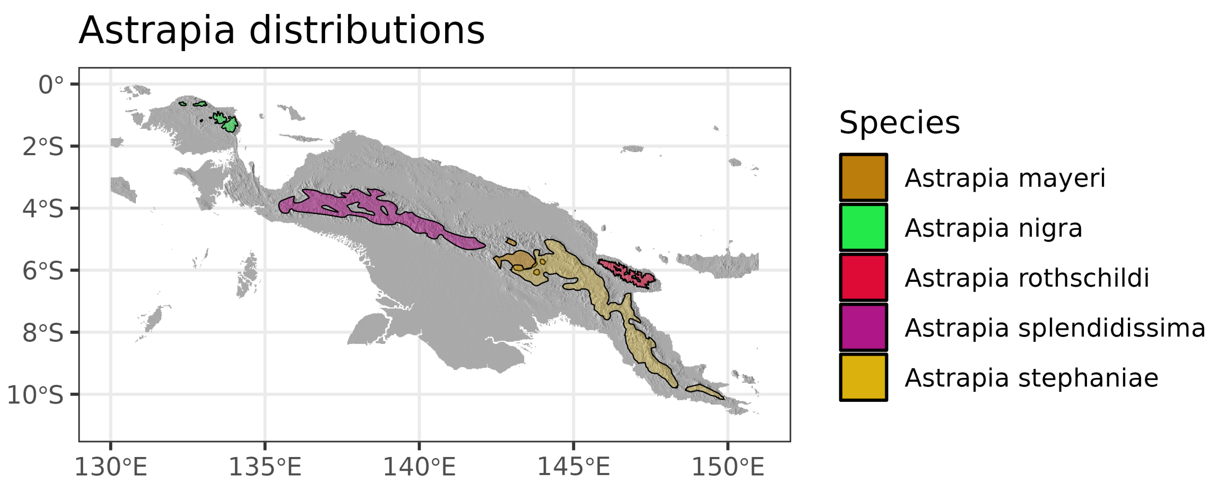

Figure S2. Species distributions for all *Astrapia* species. Distribution data obtained from IUCN.

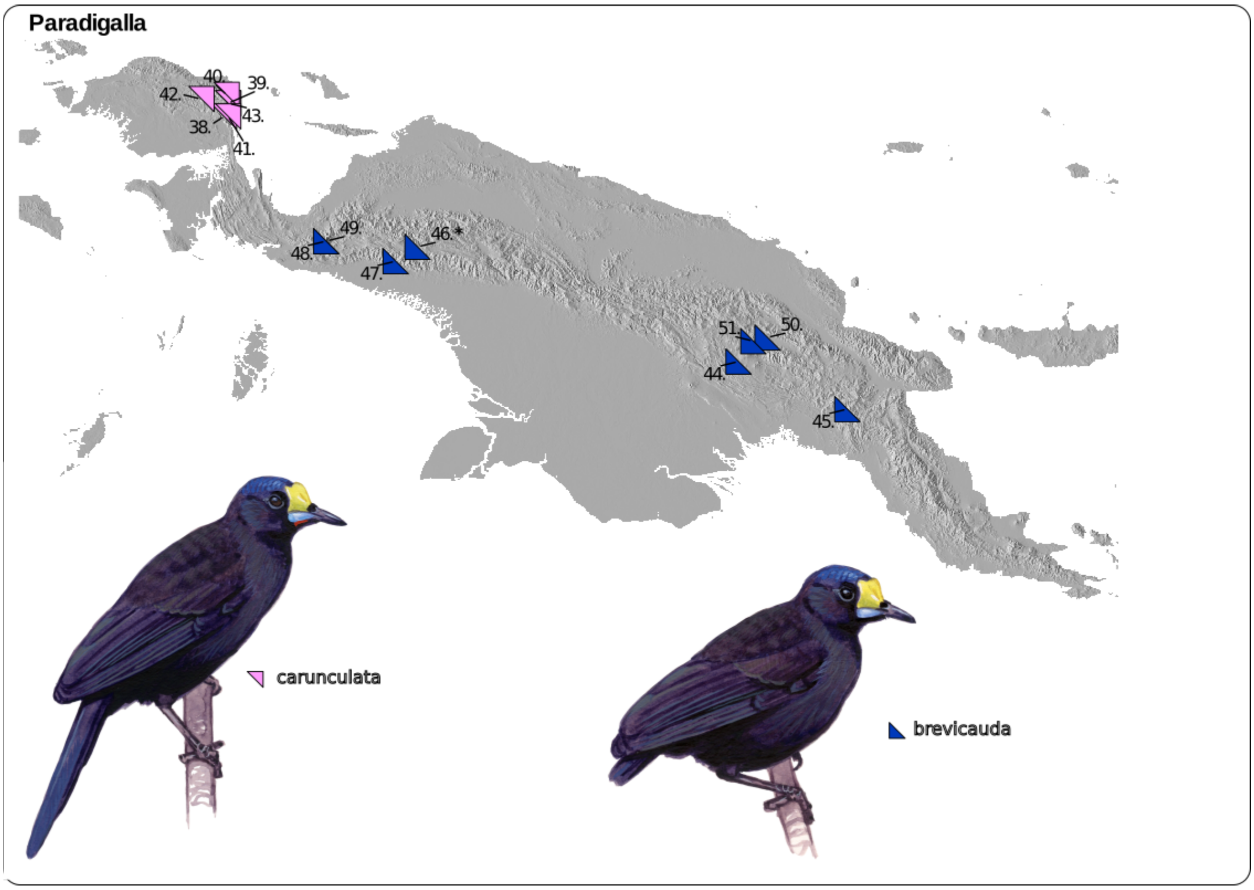

Figure S3. Sample locations of all *Paradigalla* individuals. The numbering matches the order of the samples in figure 2.

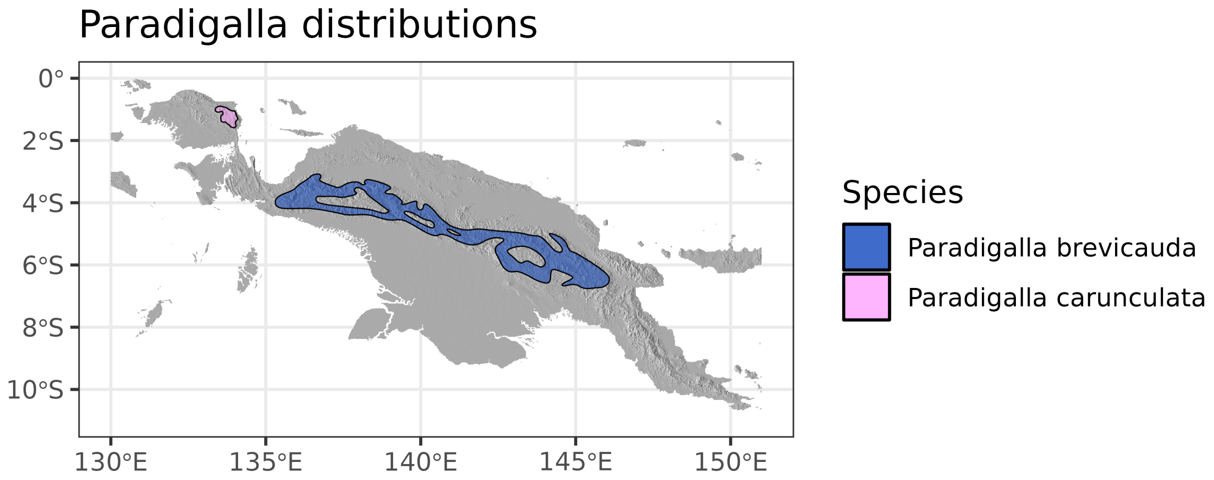

Figure S4. Species distributions for all *Paradigalla* species. Distribution data obtained from IUCN.

Pairwise Fst

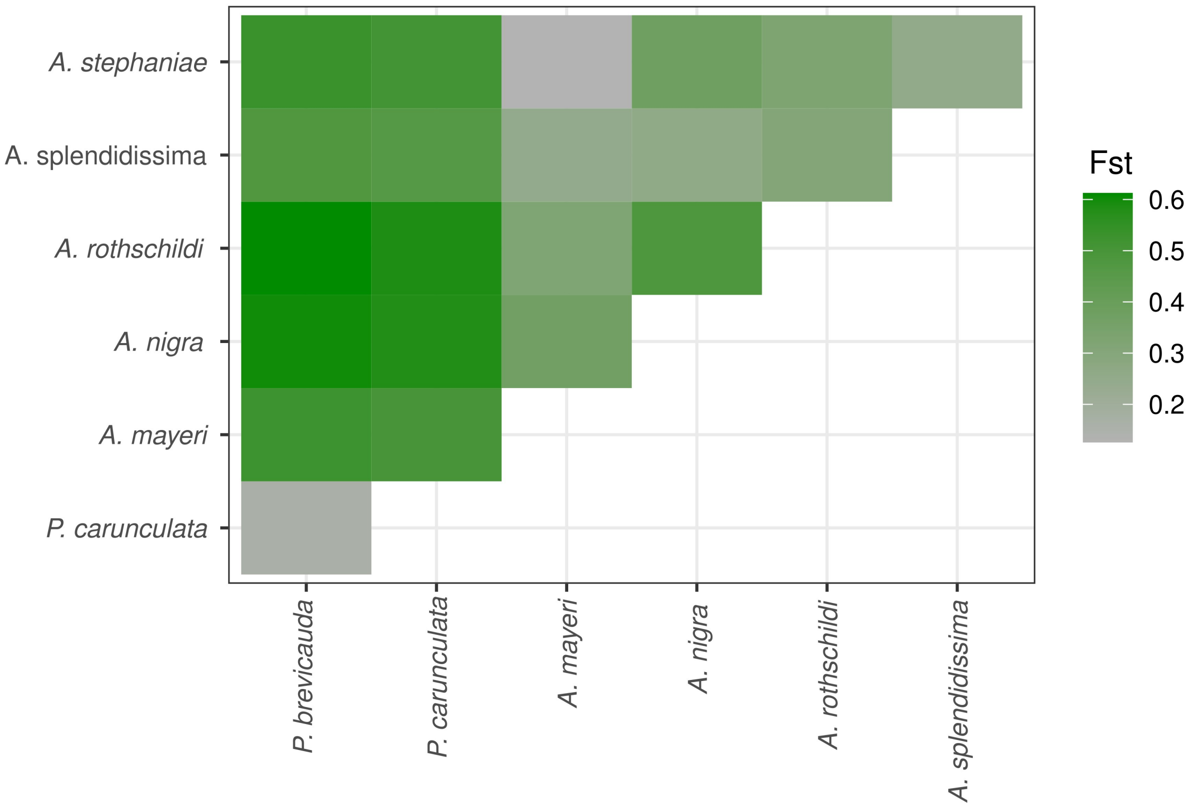

Figure S5. Pairwise fixation index (Fst) differences. All recent hybrids were excluded from the analysis. The least amount of differences are found between the two *Paradigalla* species and the *A. stephaniae* and *A. mayeri*.

Divergence time estimates

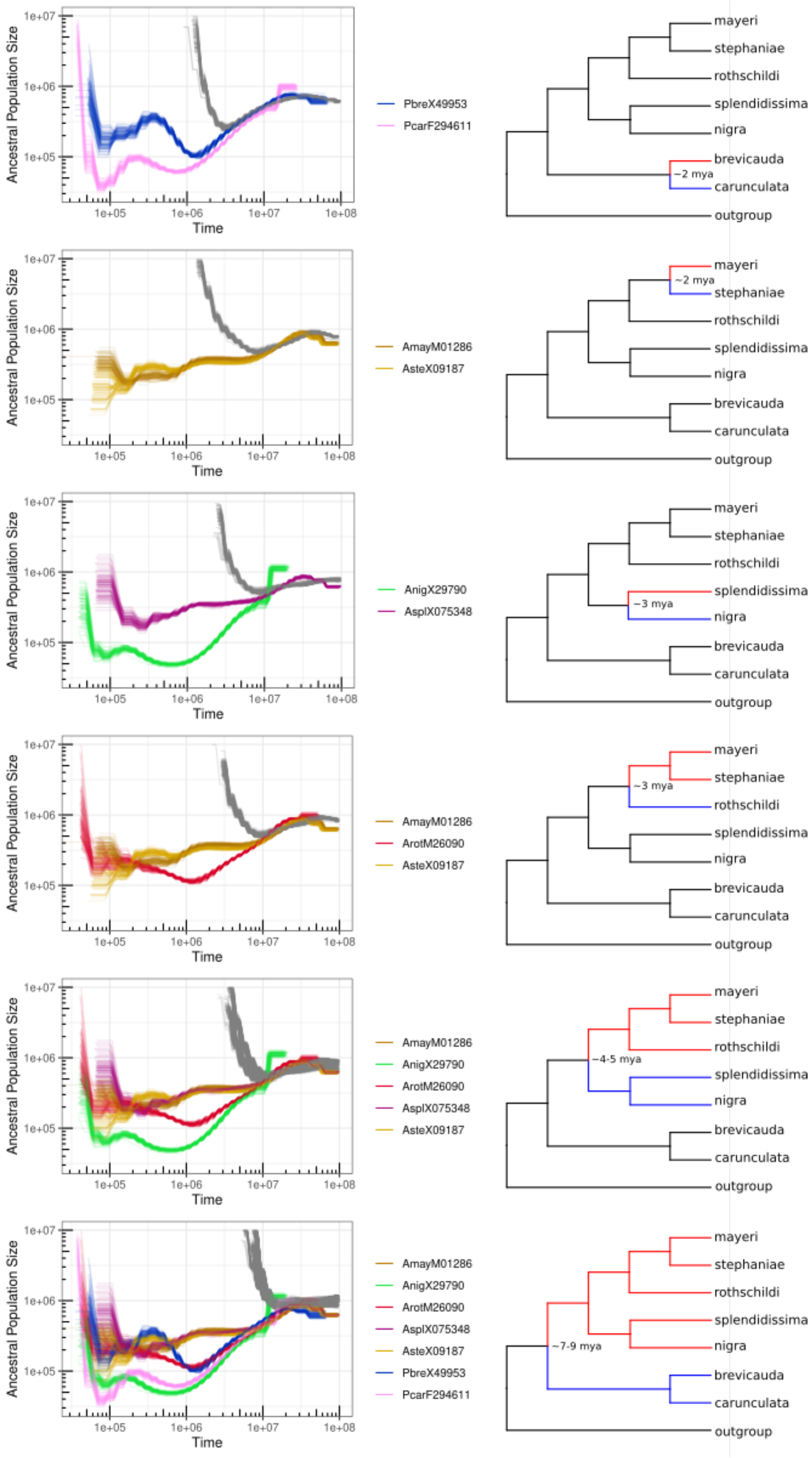

Figure S6. Divergence time estimates calculated with hPSMC with corresponding branch estimate marked on the nuclear phylogeny. hPSMC estimated based on one individual from each species. Estimated time splits derived from where the red line is 10 times the estimated ancestral population size before the initial rise.

Triangle plot

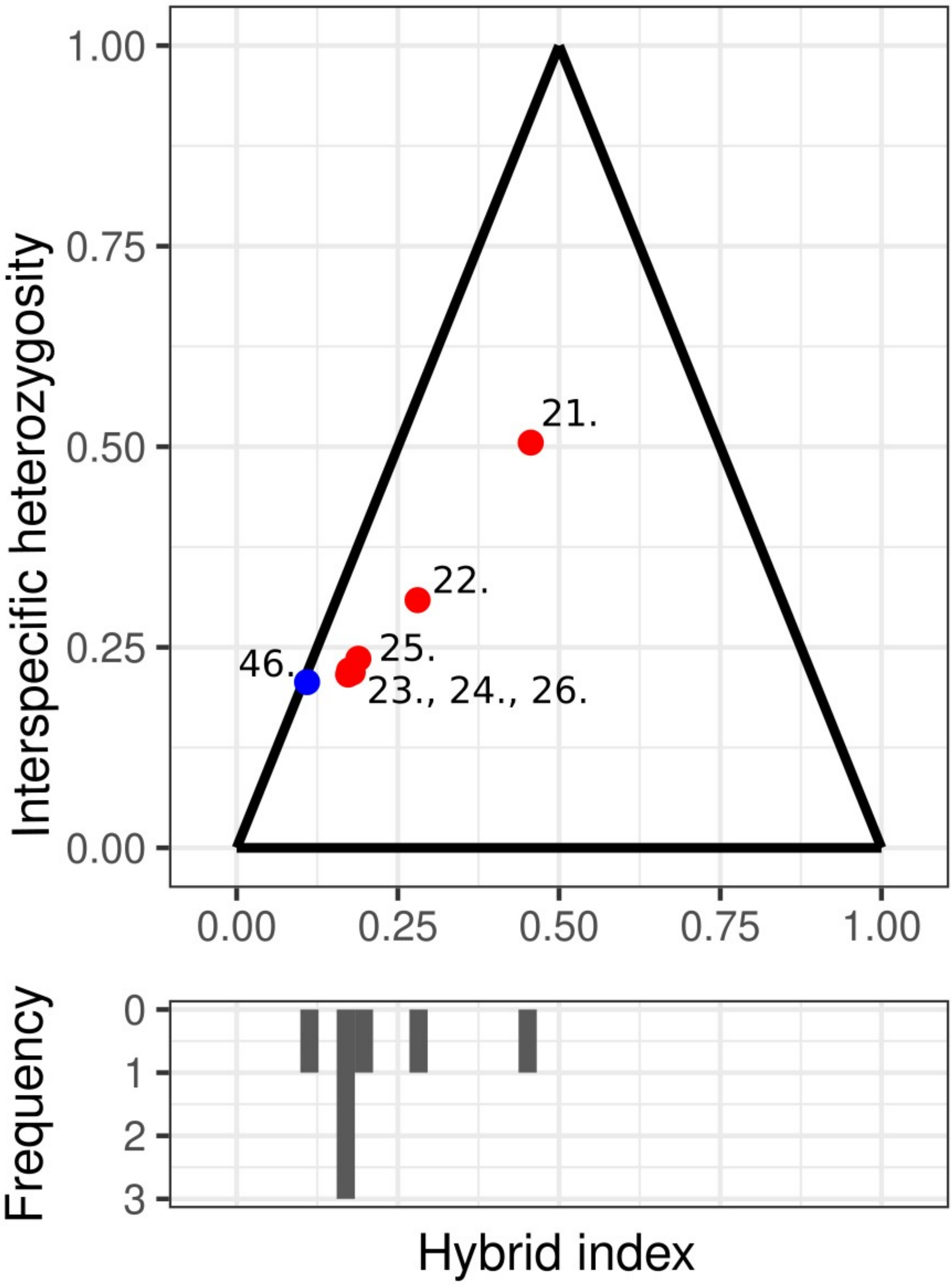

Figure S7. Interspecific heterozygosity on the y axis and proportion of parental species alleles on x axis. Hybrids between *Astrapia stephaniae* and *Astrapia mayeri* in red. The blue dot is for a hybrid between *Paradigalla brevicauda* and *Astrapia splendidissima*.

Ancestry informative markers: *Astrapia mayeri* and *Astrapia stephaniae*

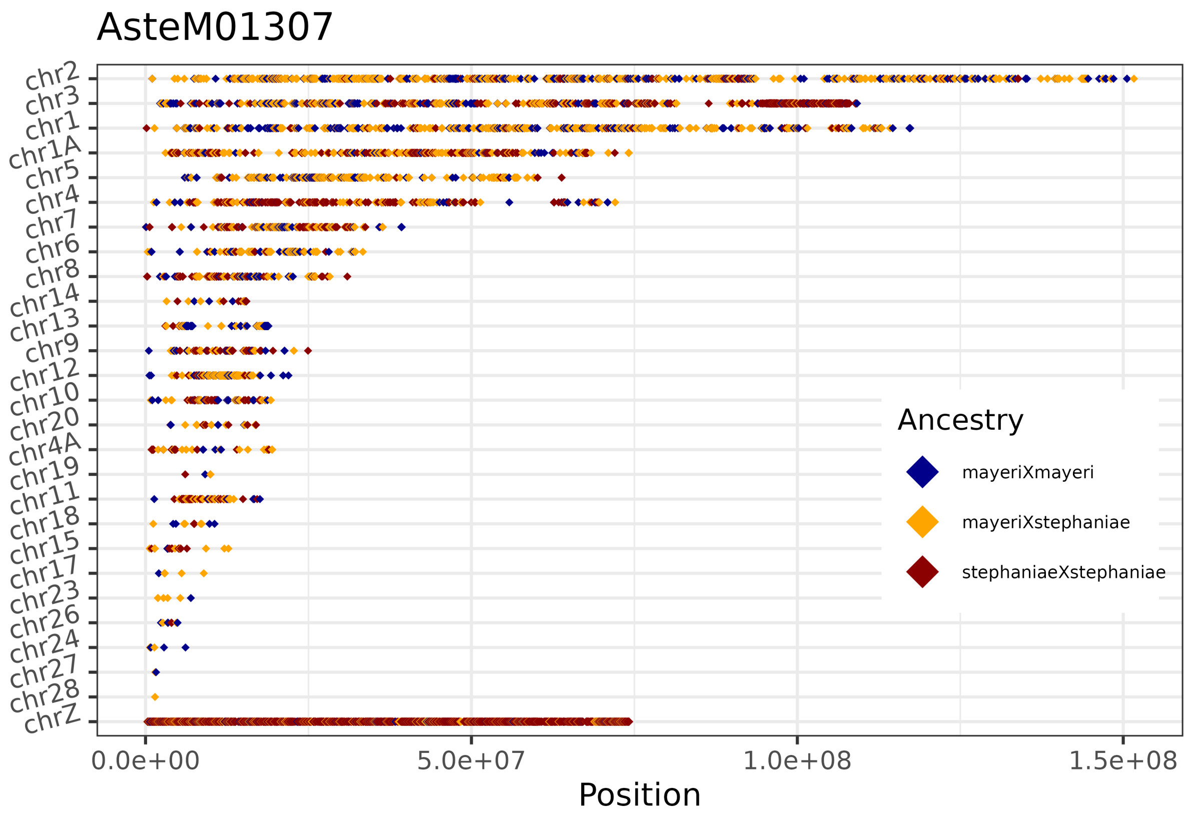

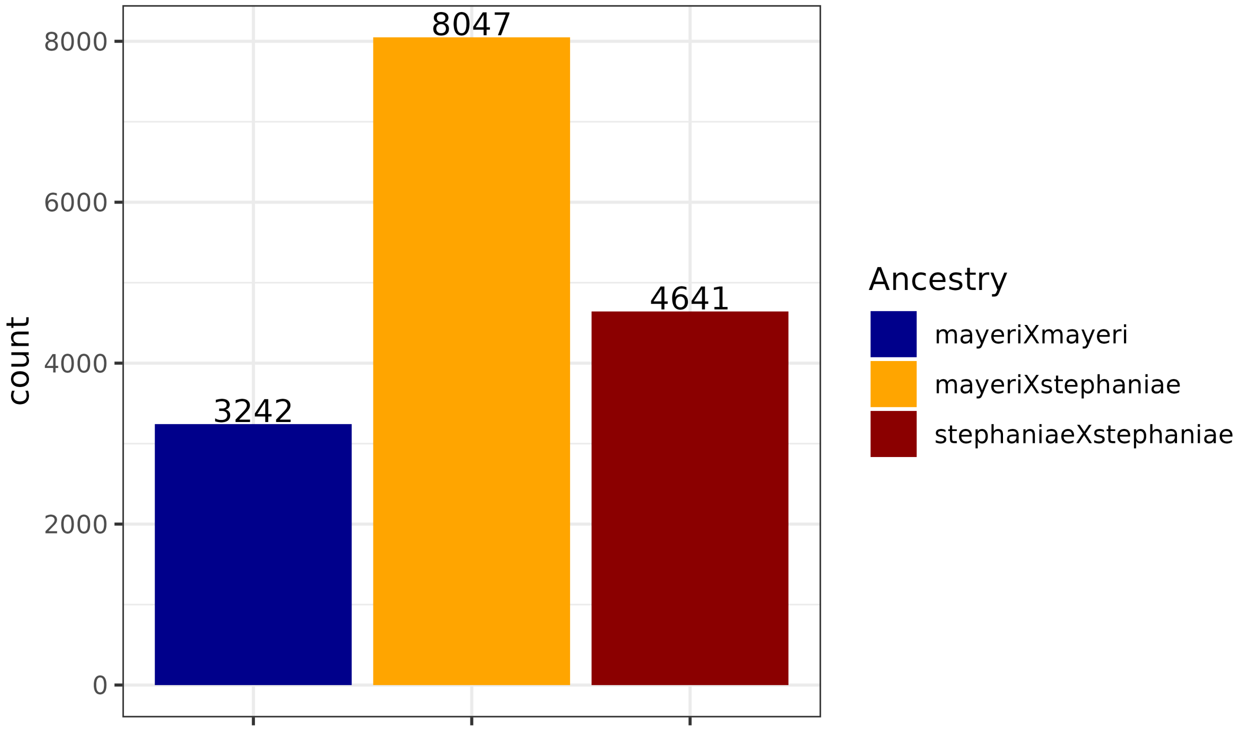

Figure S8. AIMs along autosomes and Z chromosome in AsteM01307. Counts of homozygous and heterozygous AIMs present on autosomes in AsteM01307 indicating it being a F2-hybrid.

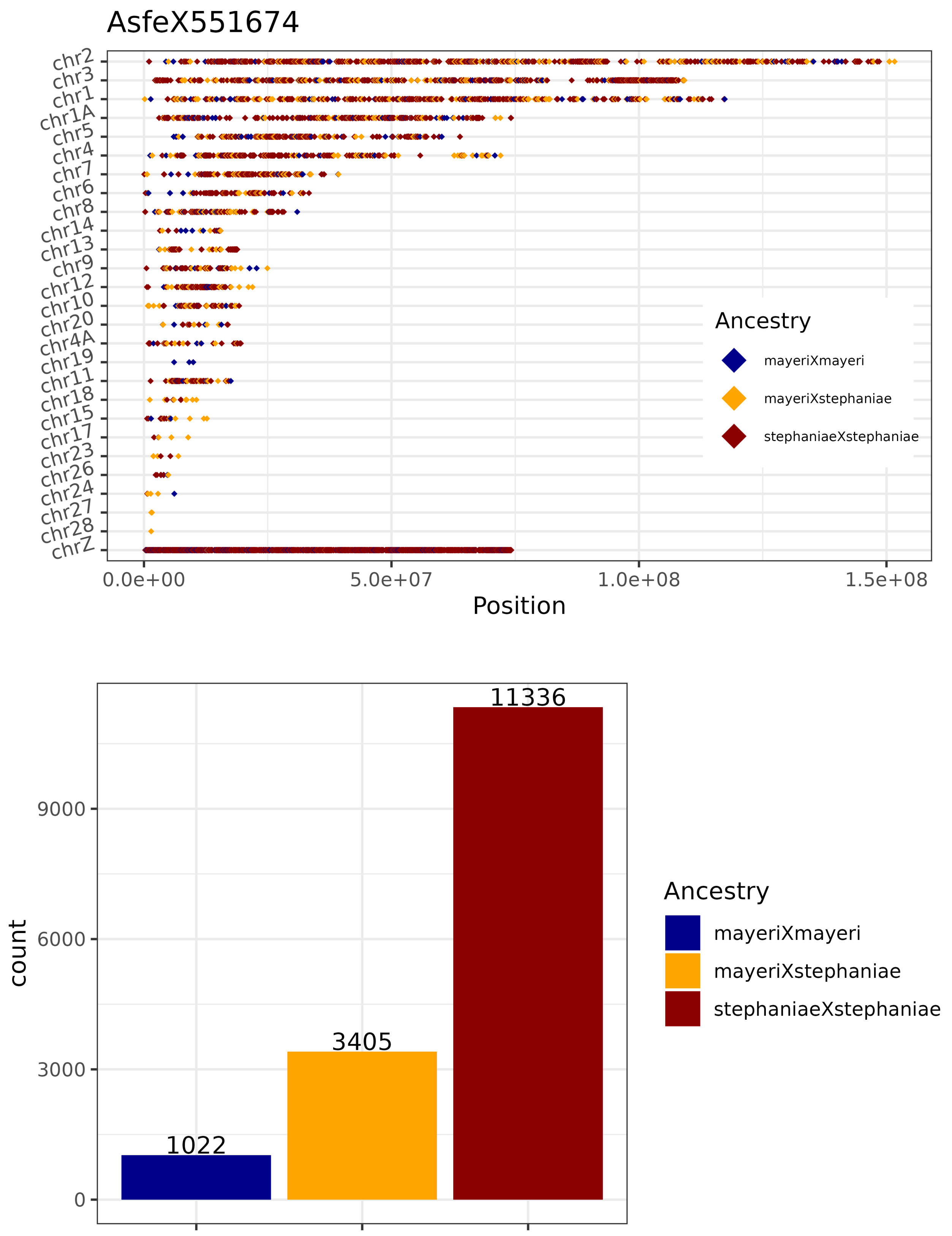

Figure S9. AIMs along autosomes and Z chromosome in AsfeX551674. Counts of homozygous and heterozygous AIMs present on autosomes in AsfeX551674.

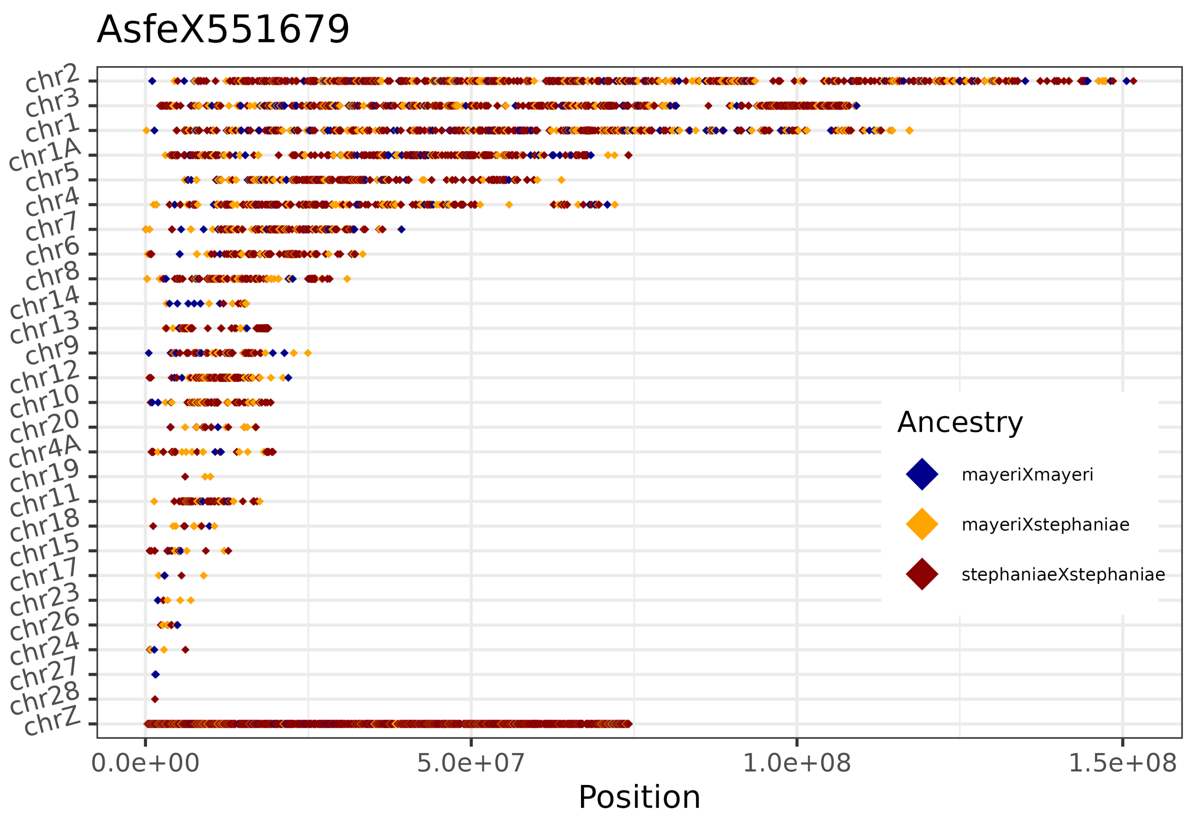

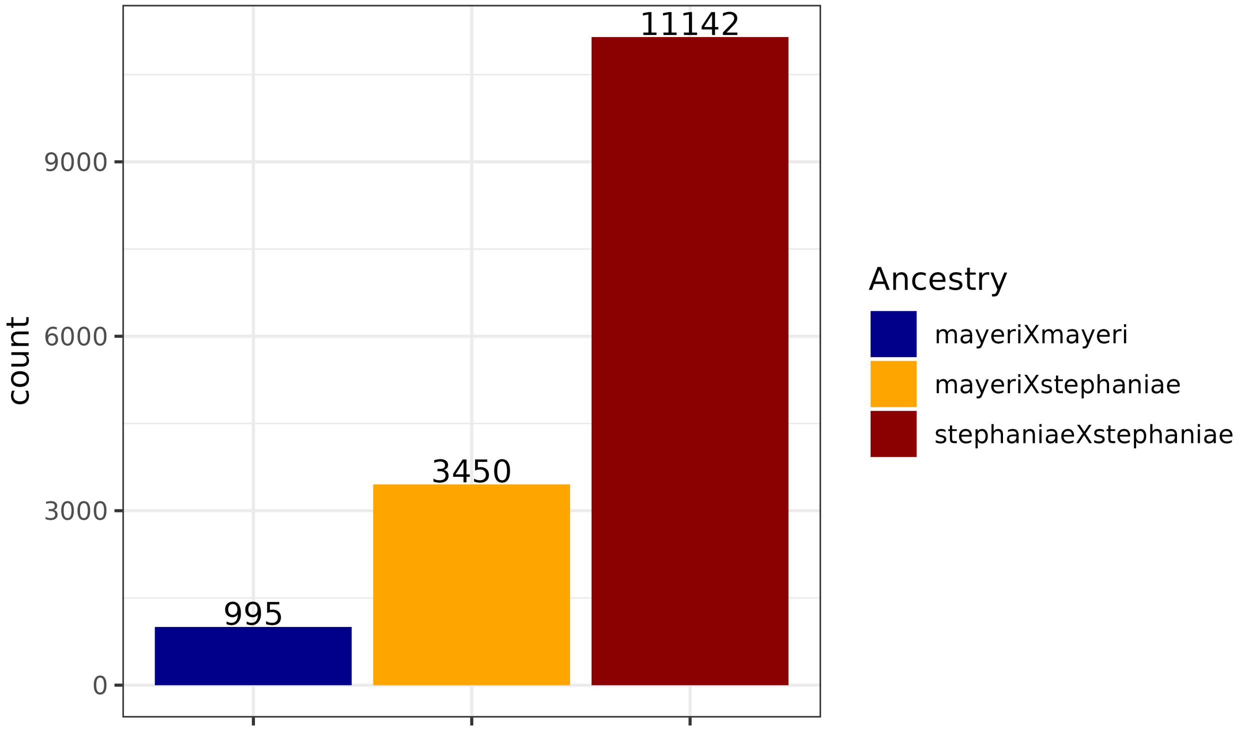

Figure S10. AIMs along autosomes and Z chromosome in AsfeX551679. Counts of homozygous and heterozygous AIMs present on autosomes in AsfeX551679.

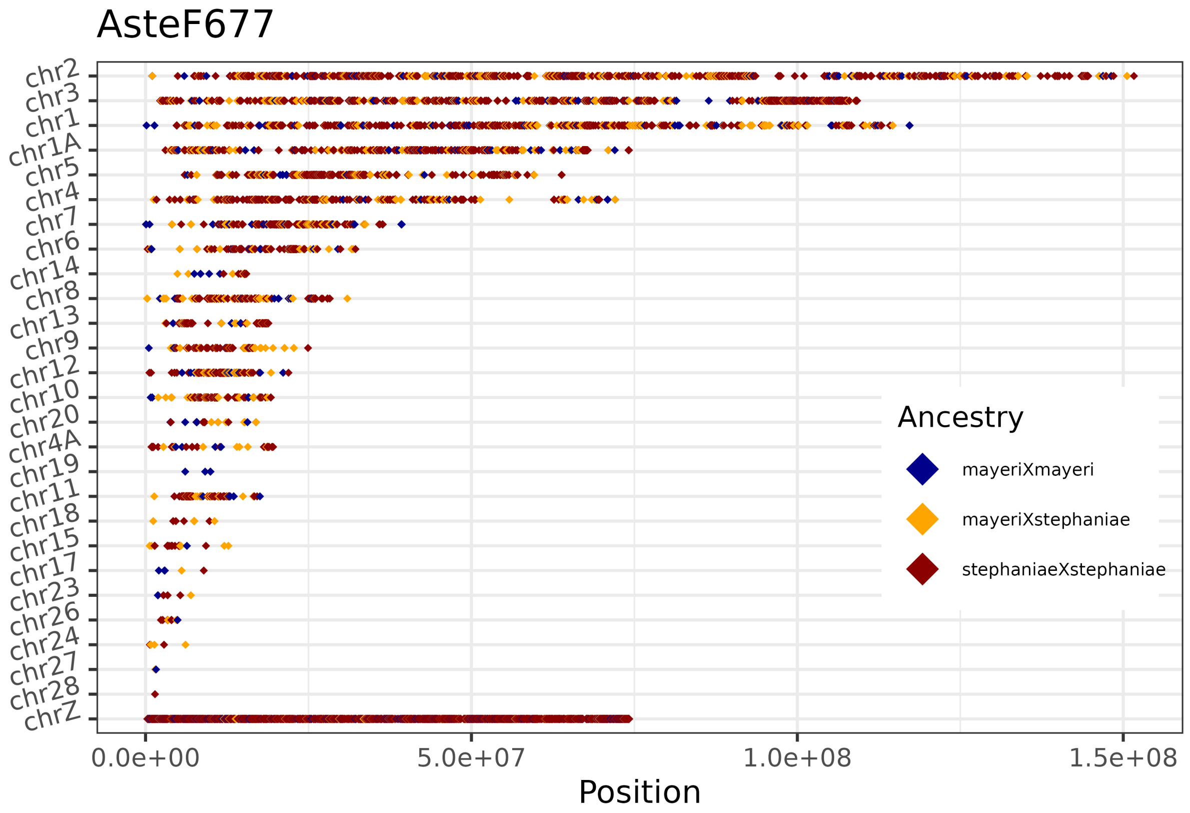

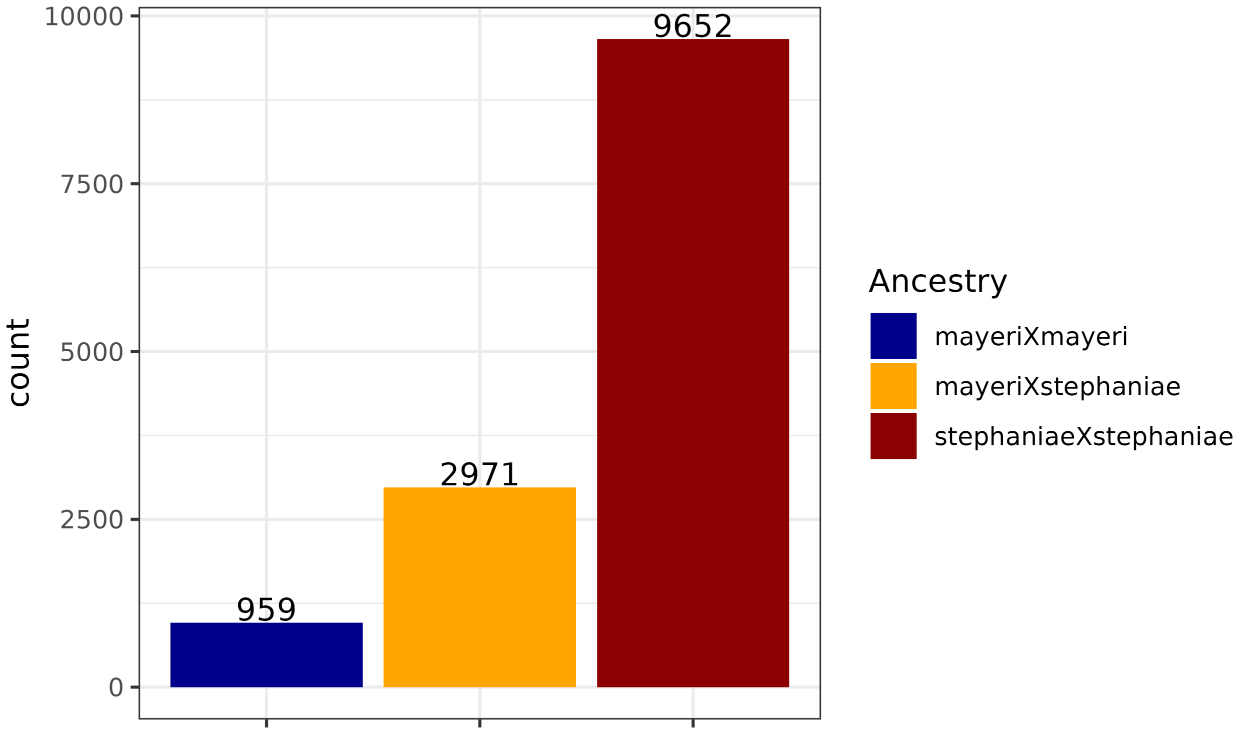

Figure S11. AIMs along autosomes and Z chromosome in AsteF677. Counts of homozygous and heterozygous AIMs present on autosomes in AsteF677.

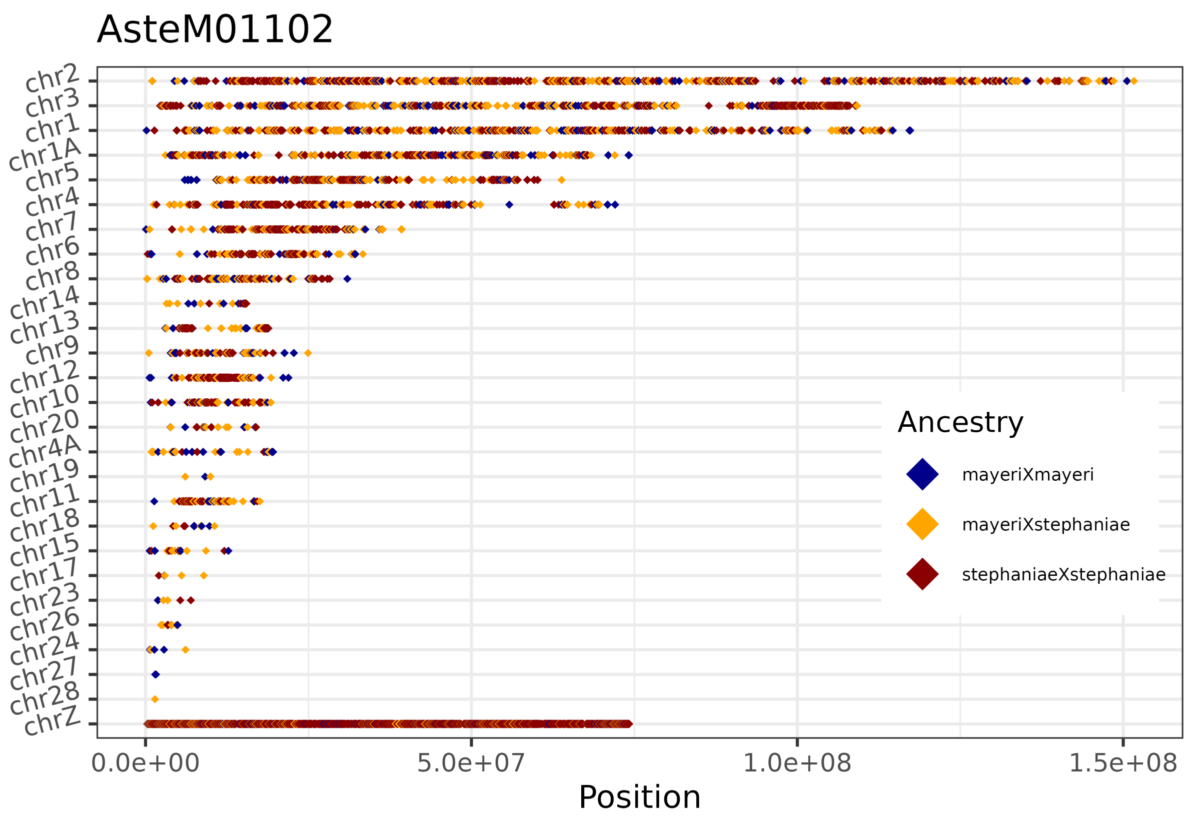

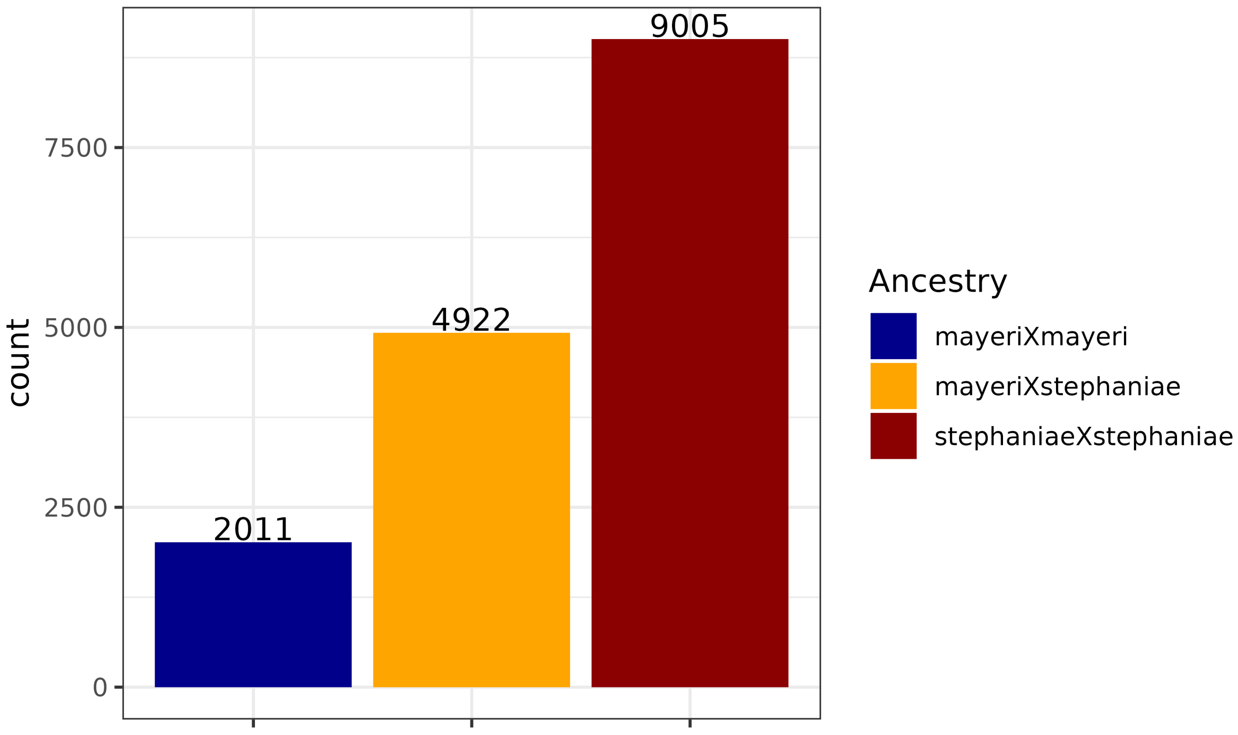

Figure S12. AIMs along autosomes and Z chromosome in AsteM01102. Counts of homozygous and heterozygous AIMs present on autosomes in AsteM01102.

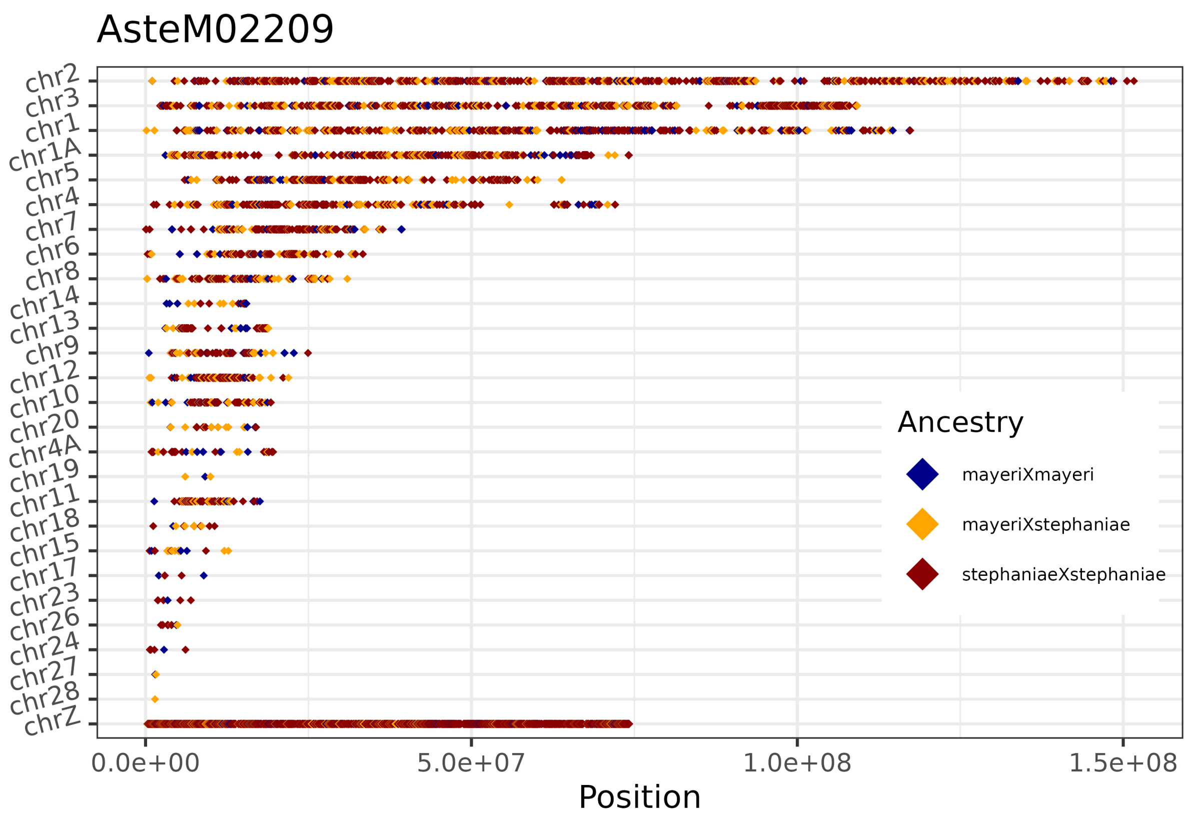

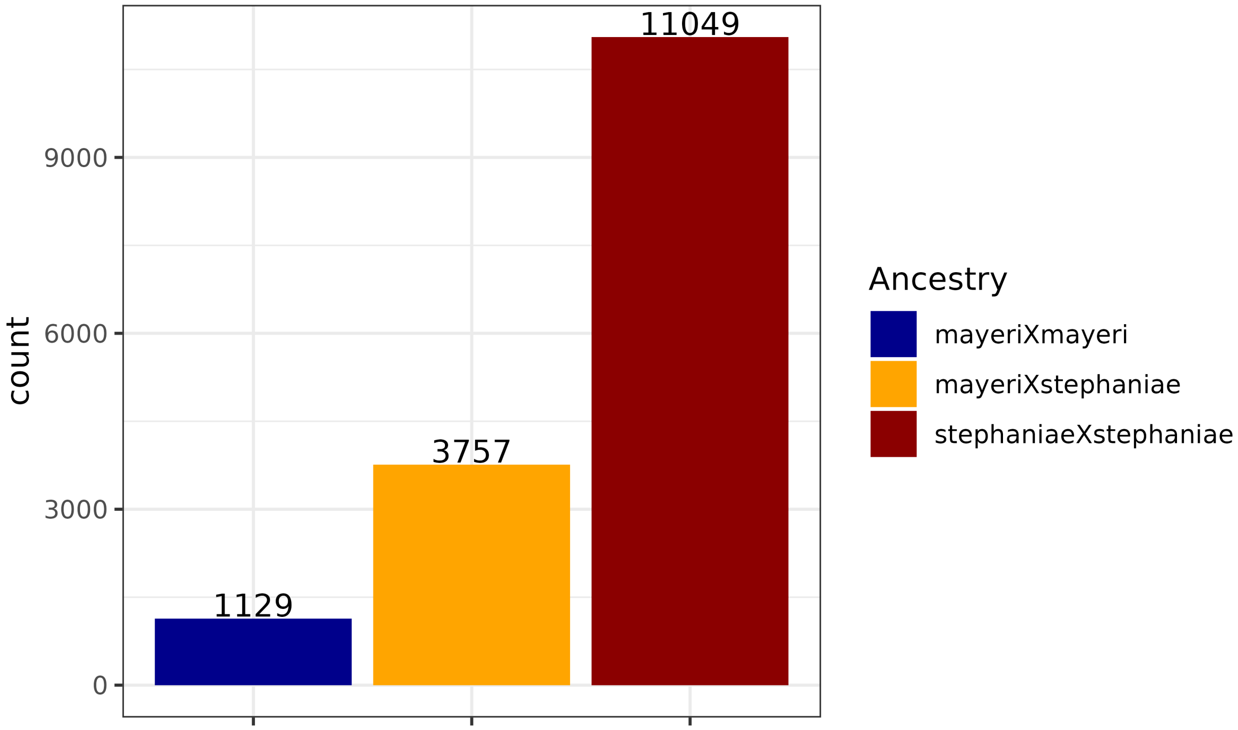

Figure S13. AIMs along autosomes and Z chromosome in AsteM02209. Counts of homozygous and heterozygous AIMs present on autosomes in AsteM02209.

Ancestry informative markers: *Astrapia splendidissima* and *Paradigalla brevicauda*

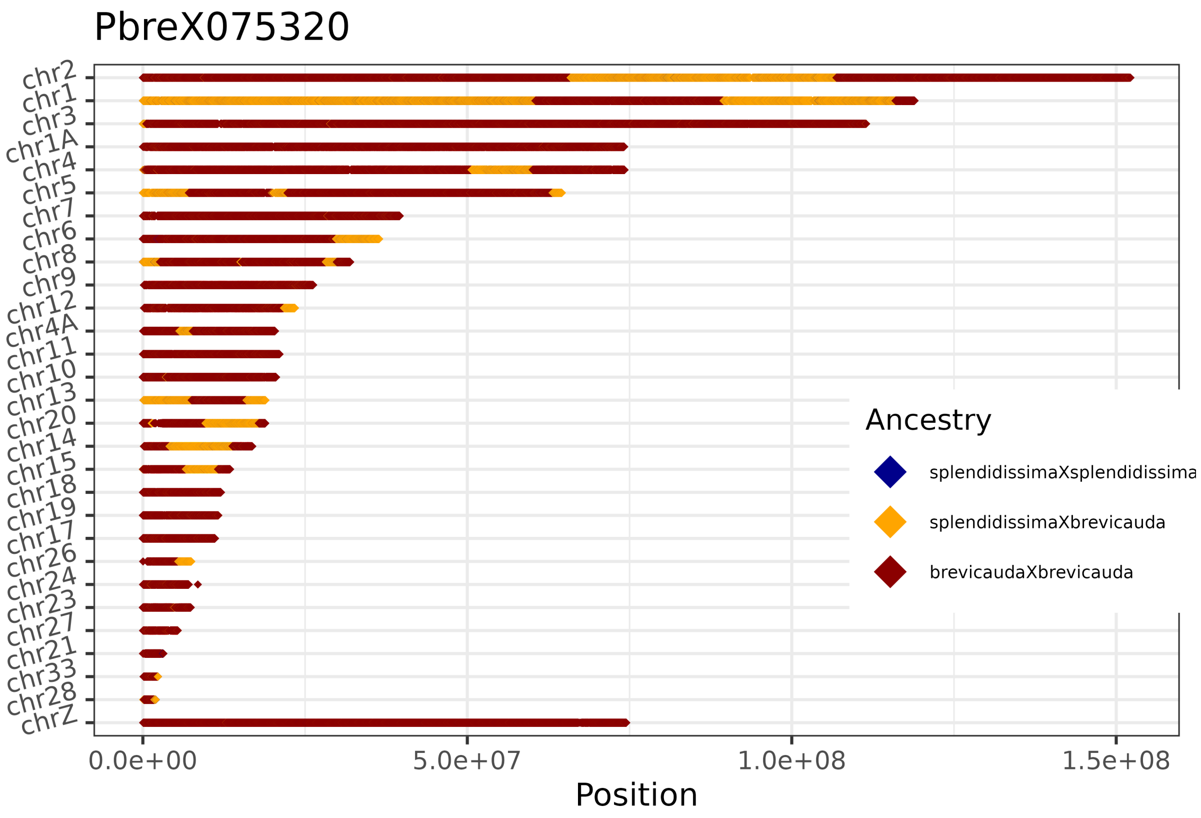

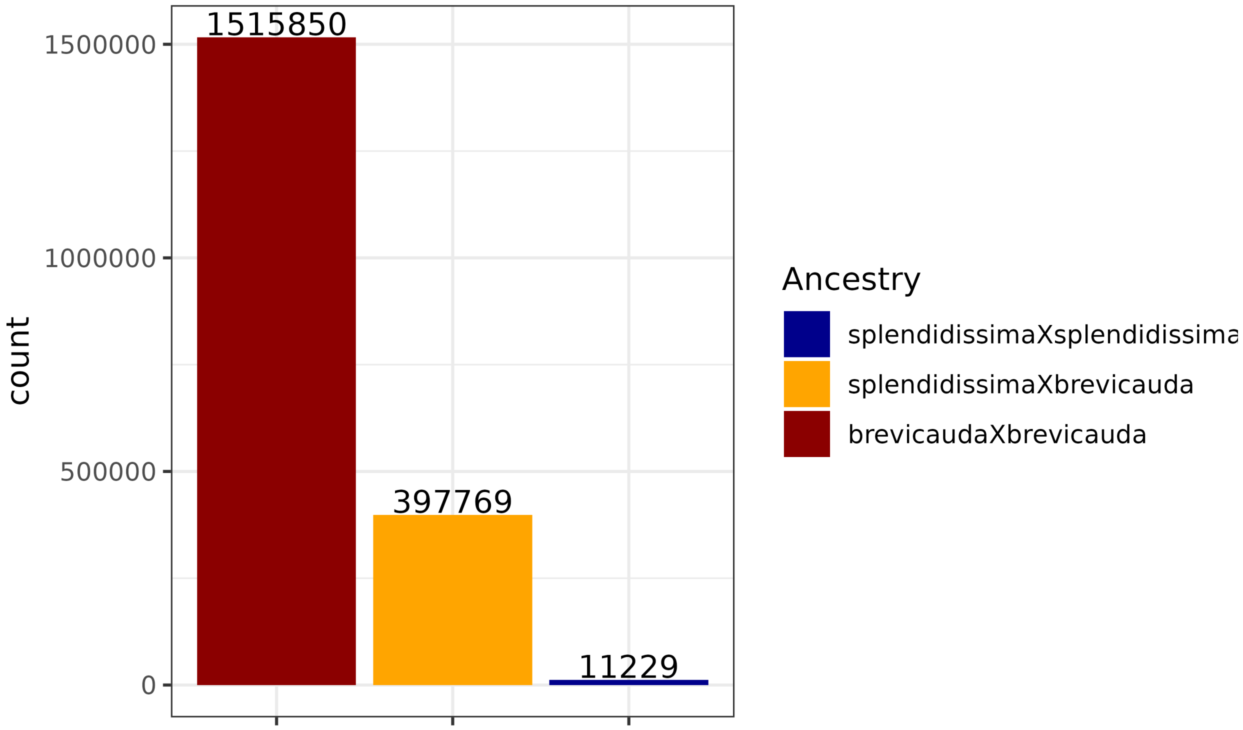

Figure S14. AIMs along autosomes and Z chromosome in PbreX075320. Counts of homozygous and heterozygous AIMs present on autosomes in PbreX075320.

AncestryPainter: *Astrapia splendidissima* and *Paradigalla brevicauda*

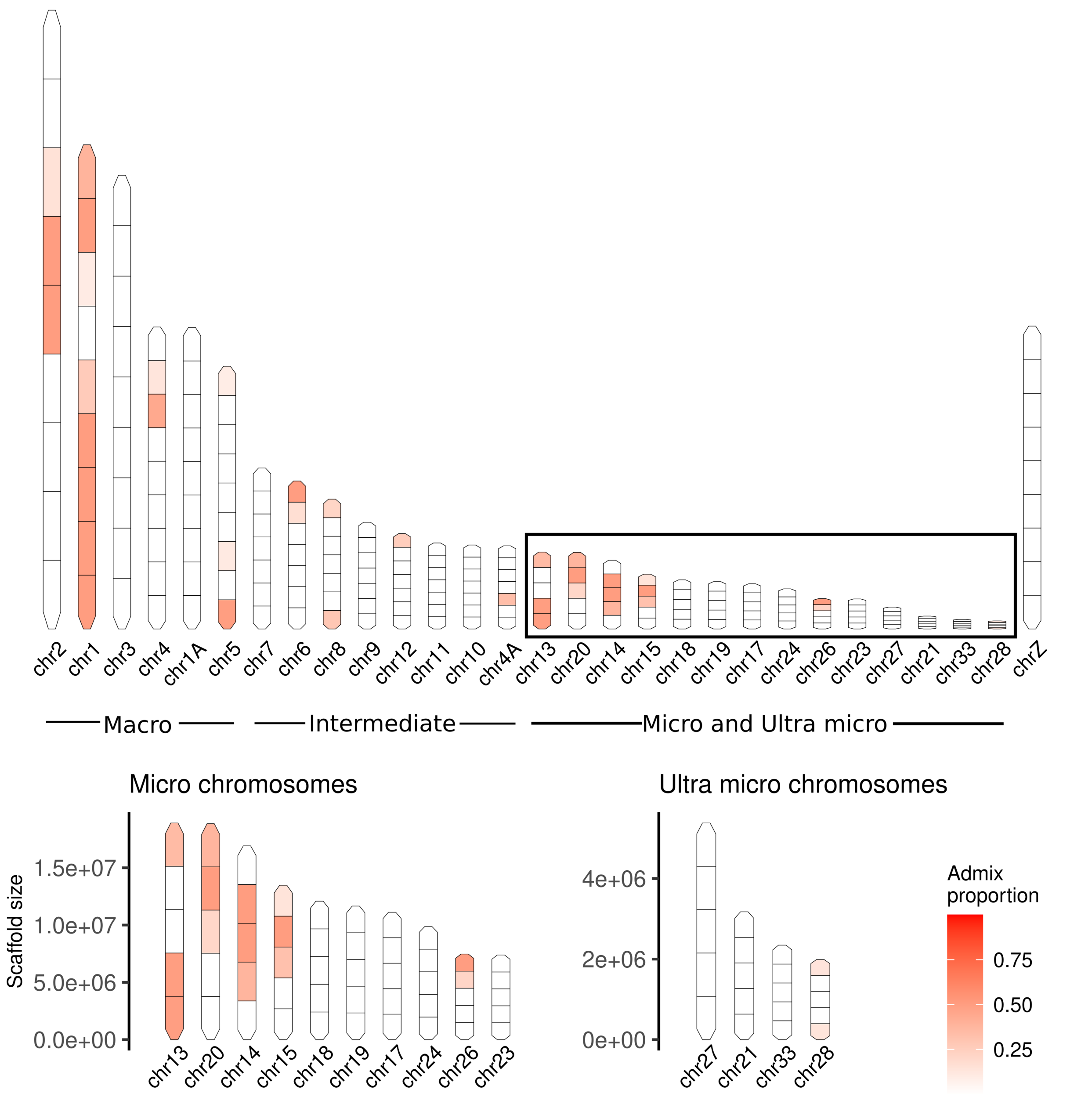

Figure S15. Admixture proportions across different regions of the chromosomes in PbreX075320, a hybrid between between *A. splendidissima* and *P. brevicauda*. High values indicate regions of high *A. splendidissima* content, which has been preserved through backcrossing with *P. brevicauda*.

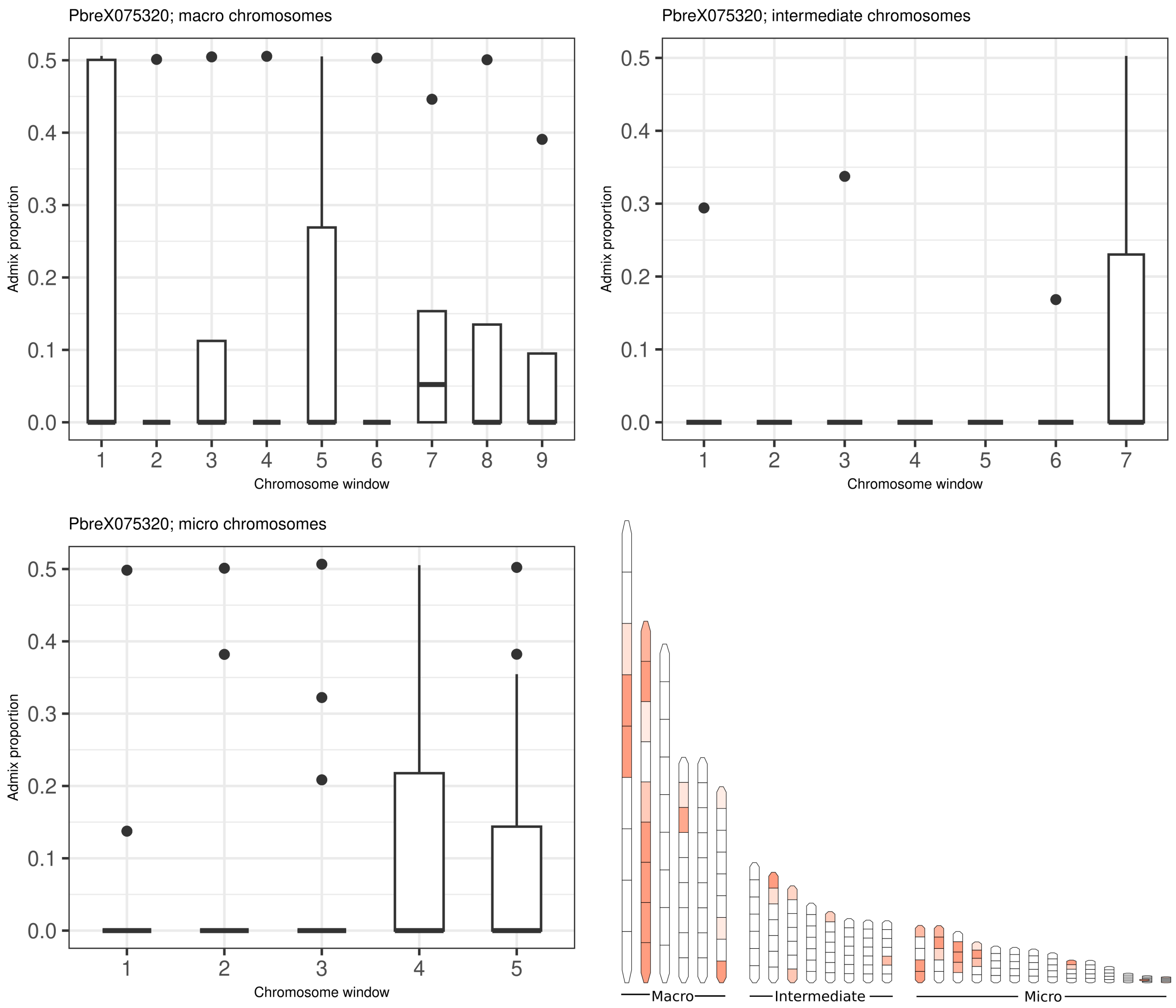

Figure S16. Introgression trends in windows distributed across chromosomes in PbreX075320, a hybrid between between *A. splendidissima* and *P. brevicauda*. A) Admixture proportion in 9 windows distributed across large chromosomes (<40Mbp), B) Admixture proportion in 7 windows distributed across intermediate chromosomes (40<20Mbp), C) Admixture proportion in 5 windows distributed across micro chromosomes (>20Mbp). D) Chromosome cartoon displaying relative size of chromosomes. Admixture proportion in windows, white low and red high. Windows not separable form incomplete lineage sorting filled with blue and are excluded from the box plots.

AncestryPainter: Individual chromosomal patterns for *Astrapia mayeri* and *Astrapia stephaniae*

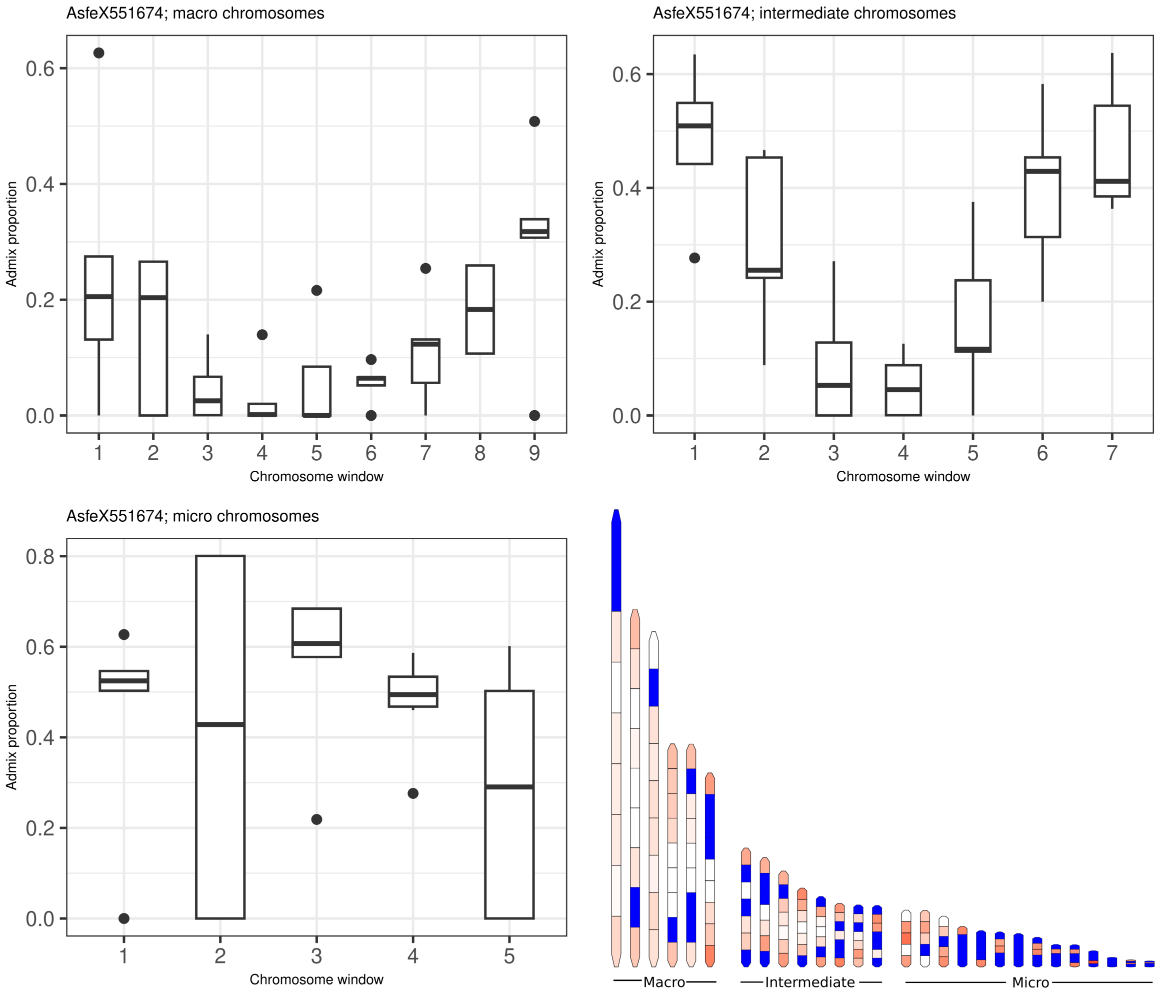

Figure S17. Introgression trends in windows distributed across chromosomes in AsfeX551674, a hybrid between between *A. mayeri* and *A. stephaniae*. A) Admixture proportion in 9 windows distributed across large chromosomes (<40Mbp), B) Admixture proportion in 7 windows distributed across intermediate chromosomes (40<20Mbp), C) Admixture proportion in 5 windows distributed across micro chromosomes (>20Mbp). D) Chromosome cartoon displaying relative size of chromosomes. Admixture proportion in windows, white low and red high. Windows not separable form incomplete lineage sorting filled with blue and are excluded from the box plots.

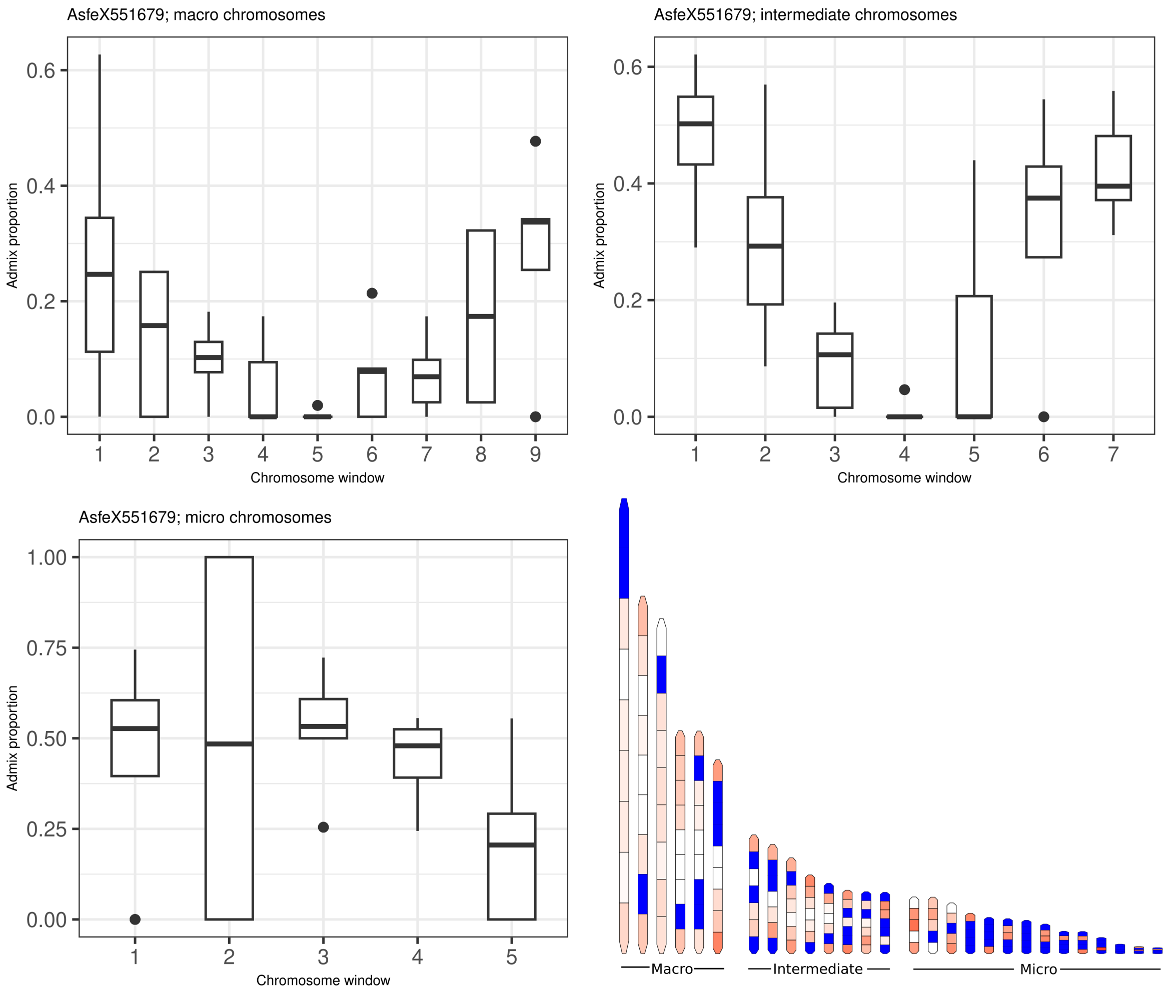

Figure S18. Introgression trends in windows distributed across chromosomes in AsfeX551679, a hybrid between between *A. mayeri* and *A. stephaniae*. A) Admixture proportion in 9 windows distributed across large chromosomes (>40Mbp), B) Median admixture proportion across the five backcrossing hybrids in 7 windows distributed across intermediate chromosomes (< 40 Mbp and >20Mbp), C) Median admixture proportion across the five backcrossing hybrids in 5 windows distributed across micro chromosomes (<20Mbp). D) Chromosome cartoon displaying relative size of chromosomes. Admixture proportion in windows, white low and red high. Windows not separable form incomplete lineage sorting filled with blue and are excluded from the box plots.

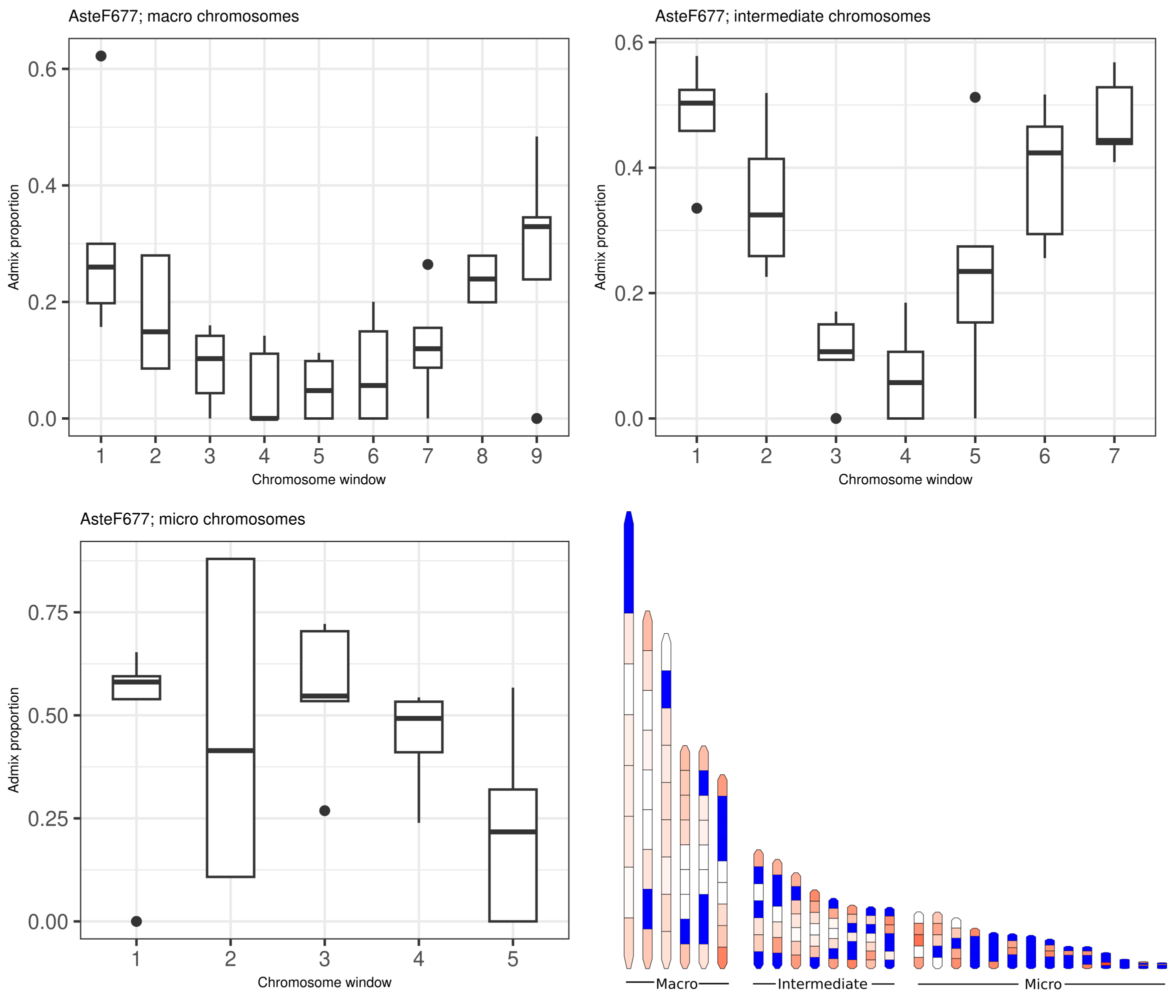

Figure S19. Introgression trends in windows distributed across chromosomes in AsteF677, a hybrid between between *A. mayeri* and *A. stephaniae*. A) Admixture proportion in 9 windows distributed across large chromosomes (>40Mbp), B) Median admixture proportion across the five backcrossing hybrids in 7 windows distributed across intermediate chromosomes (< 40 Mbp and >20Mbp), C) Median admixture proportion across the five backcrossing hybrids in 5 windows distributed across micro chromosomes (<20Mbp). D) Chromosome cartoon displaying relative size of chromosomes. Admixture proportion in windows, white low and red high. Windows not separable form incomplete lineage sorting filled with blue and are excluded from the box plots.

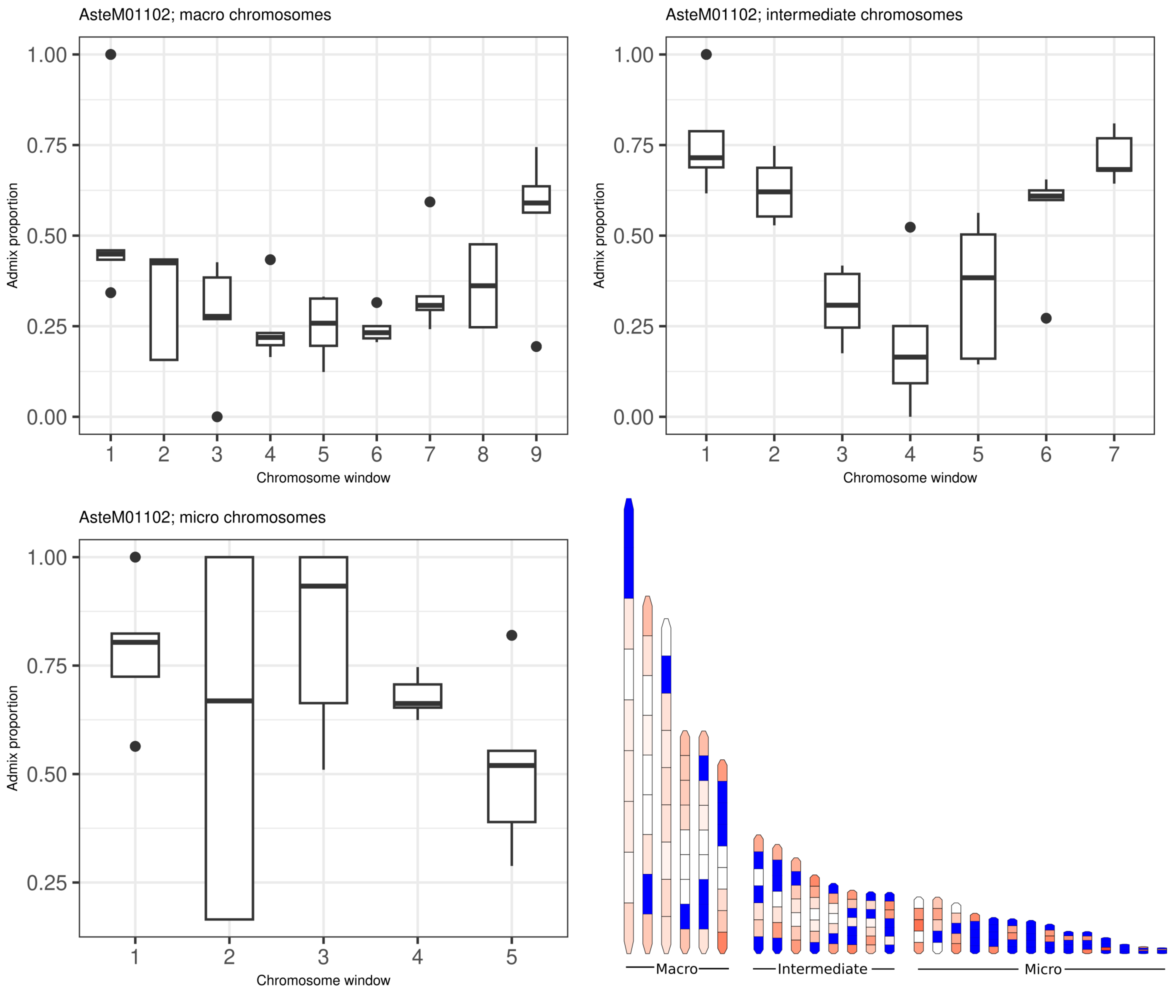

Figure S20. Introgression trends in windows distributed across chromosomes in AsteM01102, a hybrid between between *A. mayeri* and *A. stephaniae*. A) Admixture proportion in 9 windows distributed across large chromosomes (>40Mbp), B) Median admixture proportion across the five backcrossing hybrids in 7 windows distributed across intermediate chromosomes (< 40 Mbp and >20Mbp), C) Median admixture proportion across the five backcrossing hybrids in 5 windows distributed across micro chromosomes (<20Mbp). D) Chromosome cartoon displaying relative size of chromosomes. Admixture proportion in windows, white low and red high. Windows not separable form incomplete lineage sorting filled with blue and are excluded from the box plots.

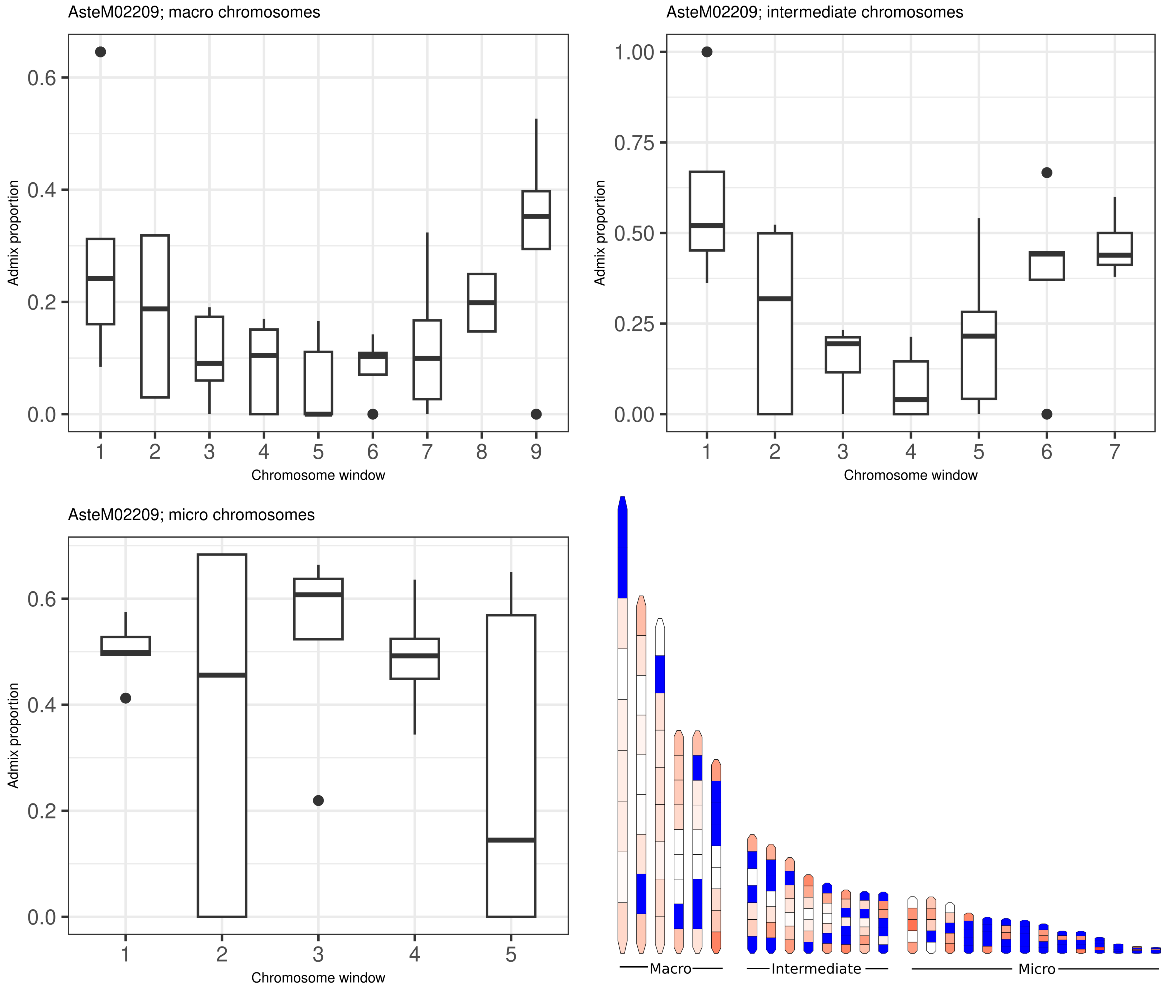

Figure S21. Introgression trends in windows distributed across chromosomes in AsteM02209, a hybrid between between *A. mayeri* and *A. stephaniae*. A) Admixture proportion in 9 windows distributed across large chromosomes (>40Mbp), B) Median admixture proportion across the five backcrossing hybrids in 7 windows distributed across intermediate chromosomes (< 40 Mbp and >20Mbp), C) Median admixture proportion across the five backcrossing hybrids in 5 windows distributed across micro chromosomes (<20Mbp). D) Chromosome cartoon displaying relative size of chromosomes. Admixture proportion in windows, white low and red high. Windows not separable form incomplete lineage sorting filled with blue and are excluded from the box plots.

Correlation between geographic distance and branch lengths

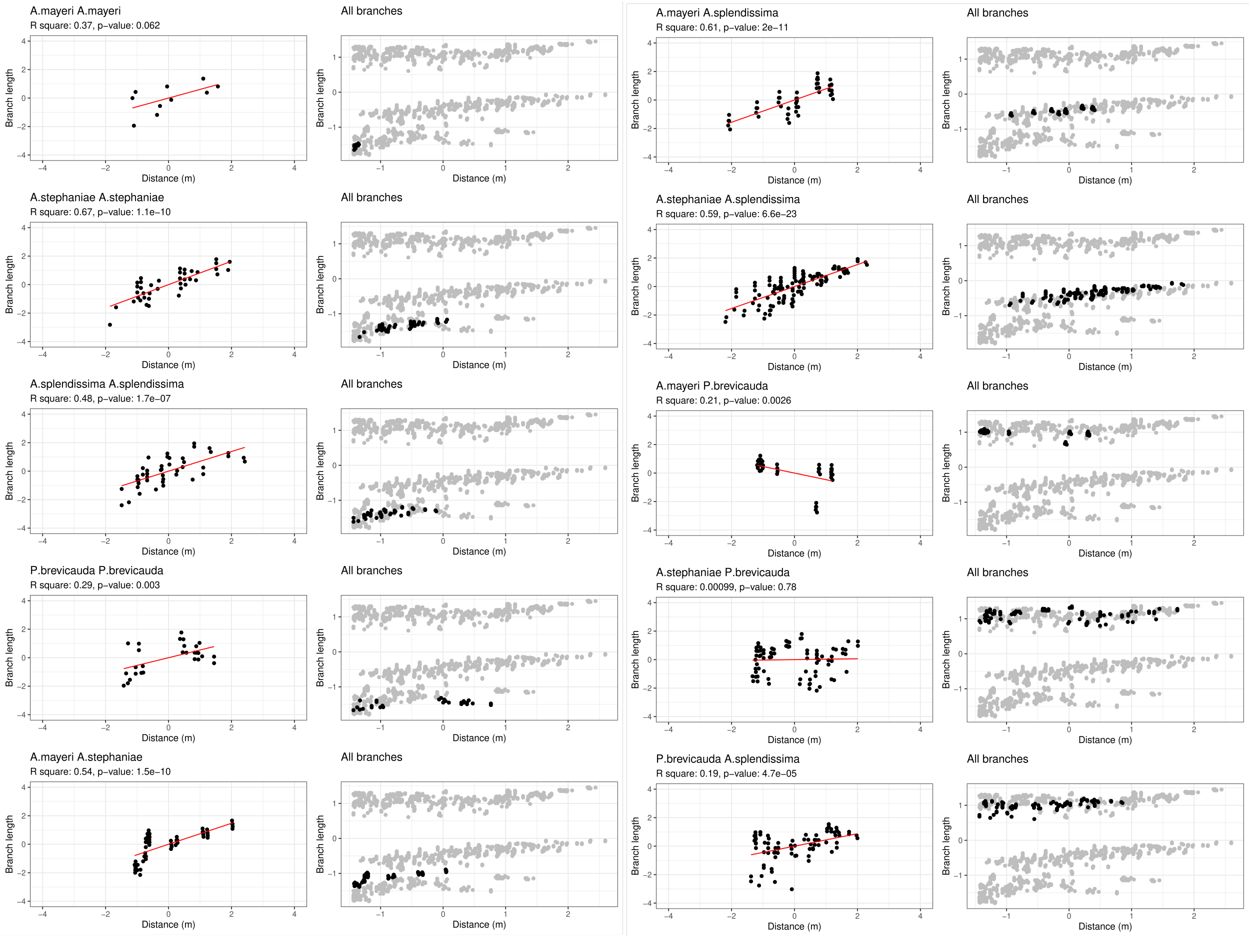

Figure S22. Correlation between pairwise geographic distance and pairwise branch lengths from maximum likelihood concatenated autosomal phylogeny for 5kb window size. All comparison including the species *A. nigra, A. rothschildi* and *P. carunculata* were removed, as these species have very small distributions that would affect correlations if meta data sample locations were imprecise. This effect is less pronounced when comparing species distributed over larger areas.

Autosomal phylogenies at different window sizes

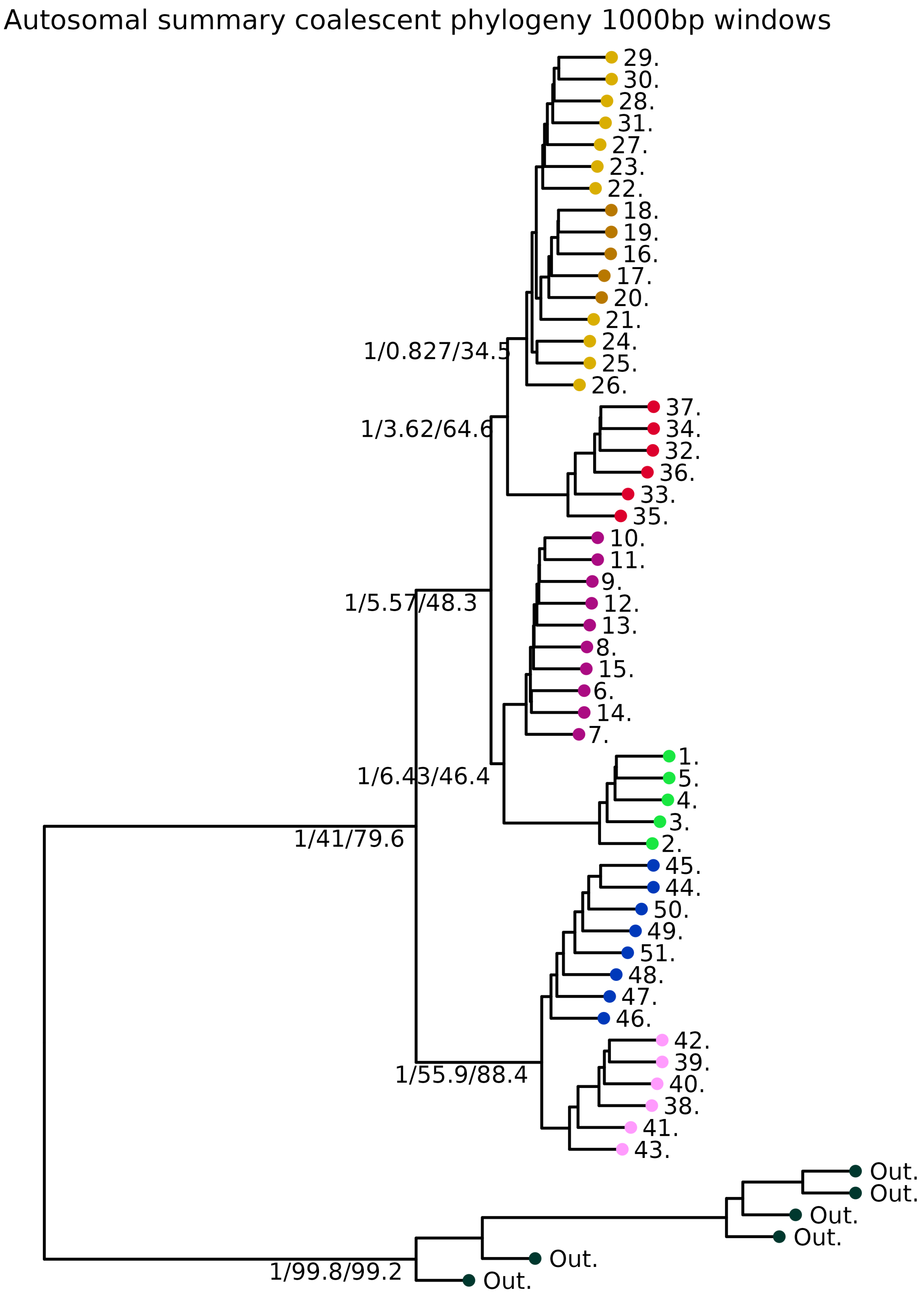

Figure S23. Autosomal summary coalescent phylogeny generated with nf-phylo using a window size of 1kb. Individuals indexed given in table S1. Support for each species clade given in bootstrap/ site concordance factors/ window concordance factors.

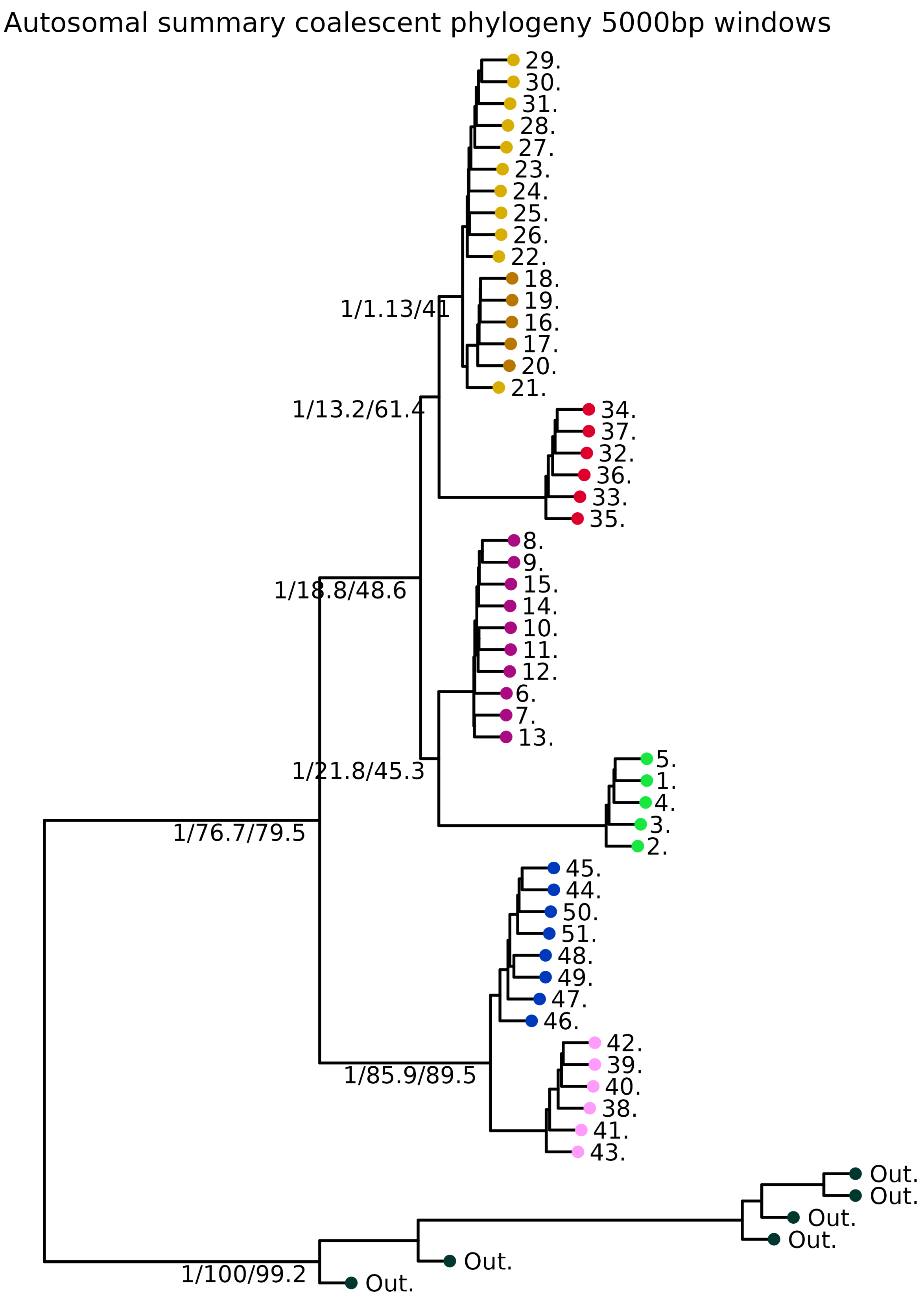

Figure S24. Autosomal summary coalescent phylogeny generated with nf-phylo using a window size of 5kb. Individuals indexed given in table S1. Support for each species clade given in bootstrap/ site concordance factors/ window concordance factors.

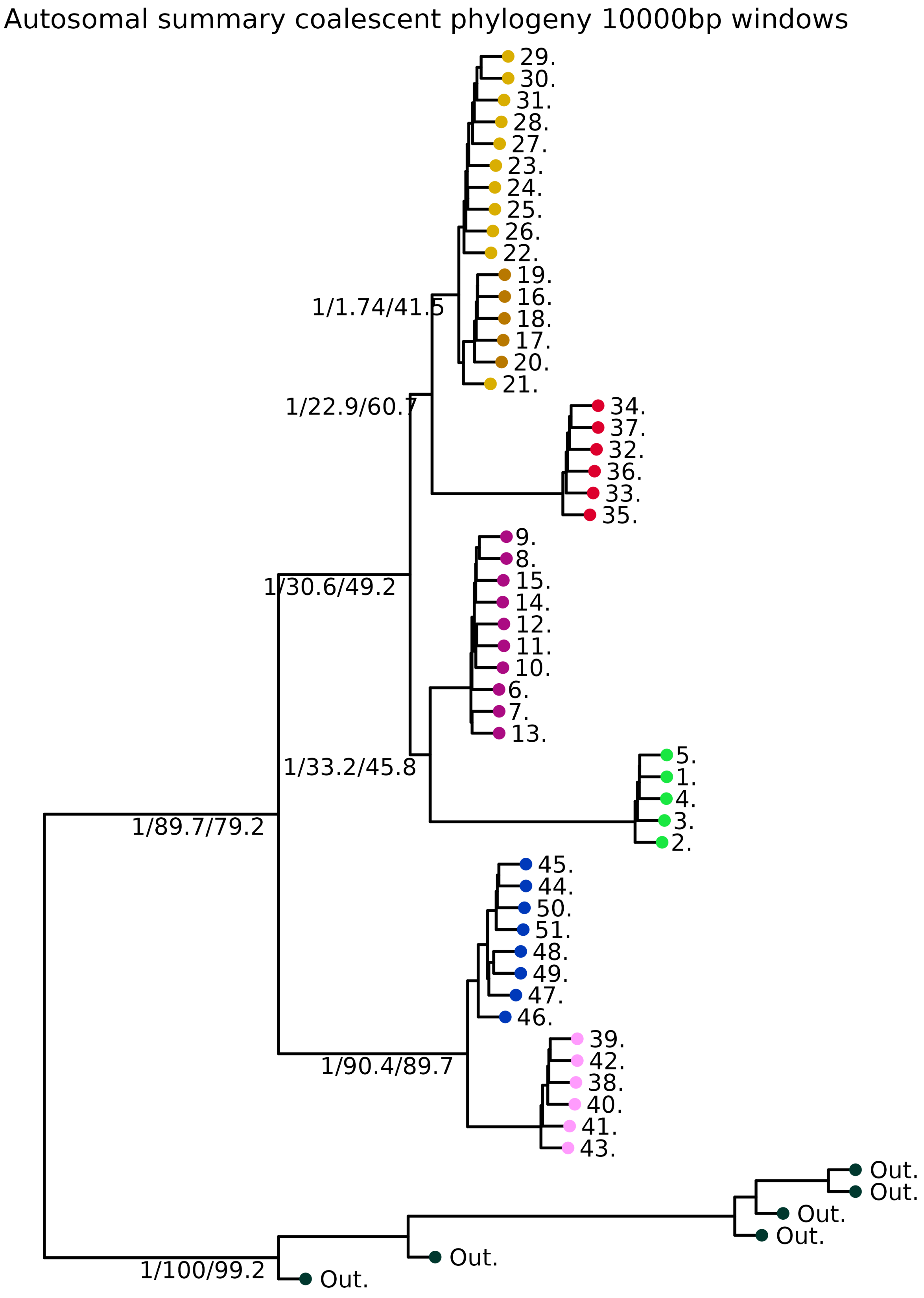

Figure S25. Autosomal summary coalescent phylogeny generated with nf-phylo using a window size of 10kb. Individuals indexed given in table S1. Support for each species clade given in bootstrap/ site concordance factors/ window concordance factors.

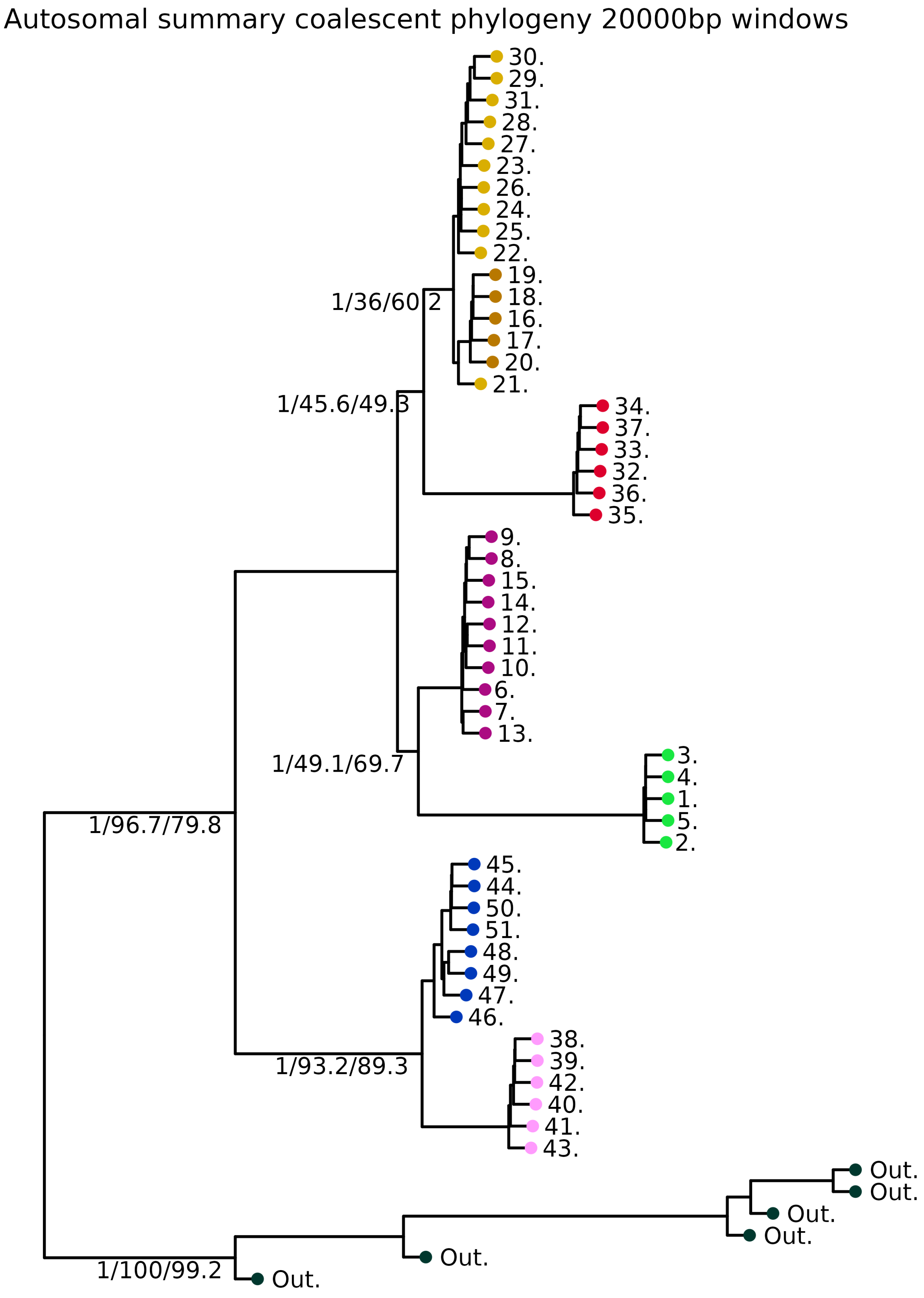

Figure S26. Autosomal summary coalescent phylogeny generated with nf-phylo using a window size of 20kb. Individuals indexed given in table S1. Support for each species clade given in bootstrap/ site concordance factors/ window concordance factors.

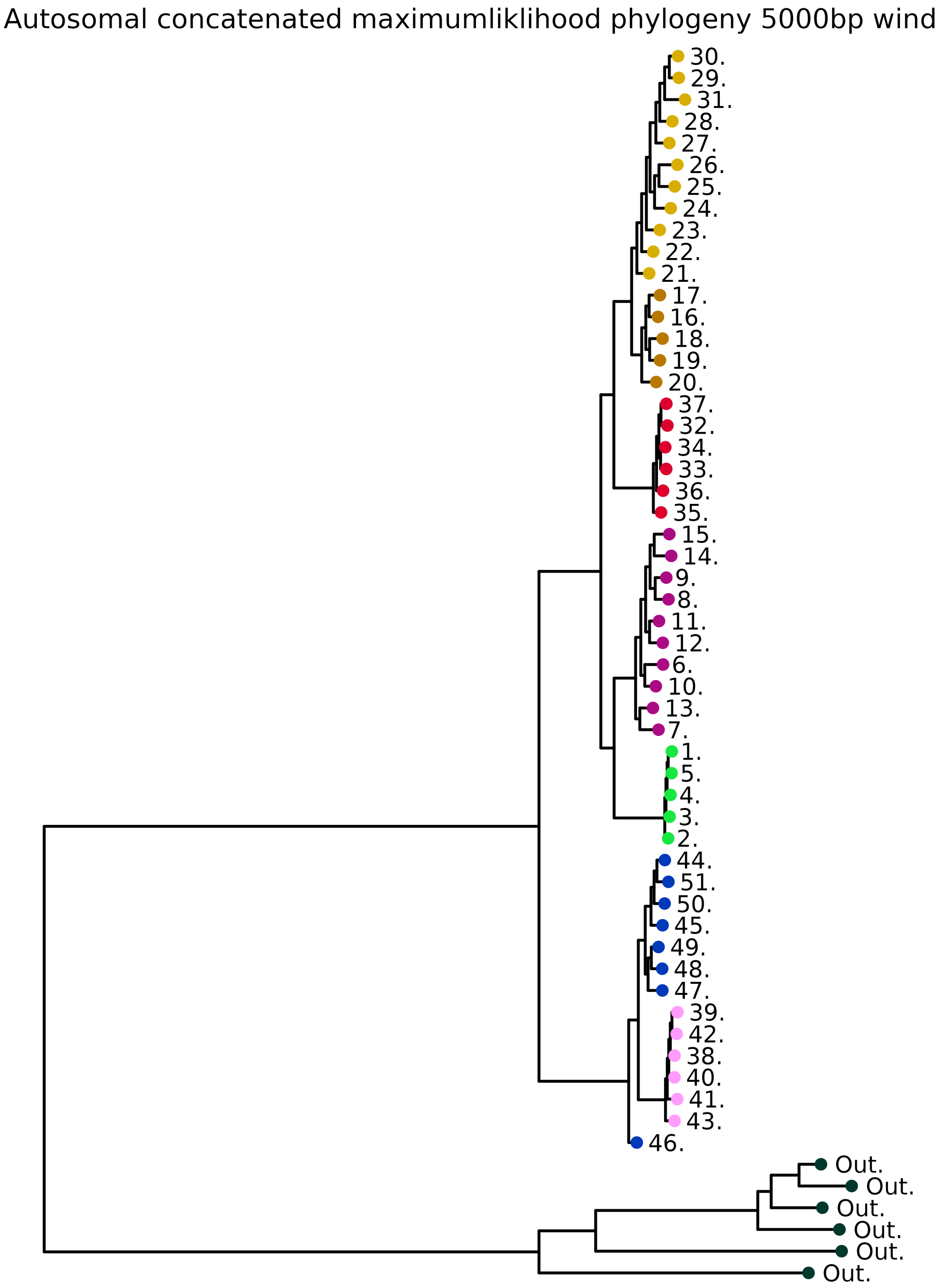

Figure S27. Concatenated maximum likelihood autosomal phylogeny generated with nf-phylo using a window size of 5kb. Individuals indexed given in table S1.

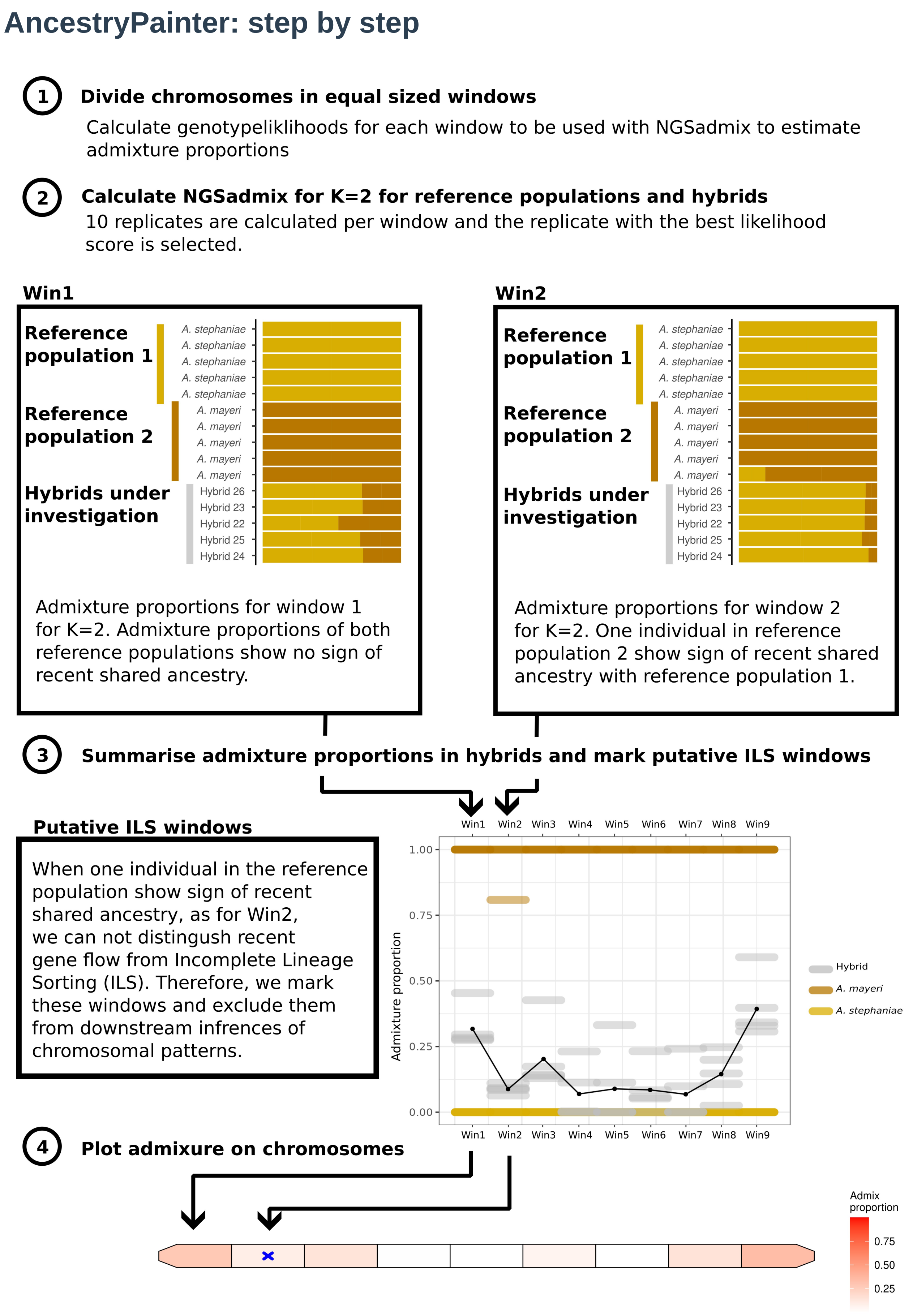

Figure S28. Cartoon explaining the different steps of AdmixturePainter and when windows are filtered out if Incomplete Lineage Sorting is assigned to a window due to shared recent reference population ancestry.

Supplemental table

Table S1. Summary of all samples included in the study. Table contains: ID, Museum registration numbers, Figure index, Genus, Species, Sex, Tissue, source, Tissue, type, Data source, lat, long, Depth.
